# Effect of myofibril passive elastic properties on the mechanical communication between motor proteins on adjacent sarcomeres

**DOI:** 10.1038/s41598-019-45772-1

**Published:** 2019-06-27

**Authors:** Takumi Washio, Seine A. Shintani, Hideo Higuchi, Seiryo Sugiura, Toshiaki Hisada

**Affiliations:** 1 UT-Heart Inc., 178-4-4 Wakashiba, Kashiwa, 277-0871 Japan; 2 Department of Biomedical Sciences, College of Life and Health Sciences, Chubu University, 1200 Matsumoto-cho Kasugai, Aichi, 487-8501 Japan; 3 Department of Physics, Graduate School of Science, University of Tokyo, 7-3-1 Hongo Bunkyo-ku, Tokyo, 113-0033 Japan; 4 Future Center Initiative, University of Tokyo, 178-4-4 Wakashiba, Kashiwa, 277-0871 Japan

**Keywords:** Computational biophysics, Deformation dynamics, Computational science

## Abstract

Rapid sarcomere lengthening waves propagate along a single muscle myofibril during spontaneous oscillatory contraction (SPOC). In asynchronous insect flight muscles, SPOC is thought to be almost completely synchronized over the entire myofibril. This phenomenon does not require Ca^2+^ regulation of the dynamics of the motor proteins, and cannot be explained simply by the longitudinal mechanical equilibrium among sarcomeres in the myofibril. In the present study, we rationalize these phenomena by considering the lateral mechanical equilibrium, in which two tensions originating from the inverse relationship between sarcomere length and lattice spacing, along with the lattice alignment, play important roles in the mechanical communication between motor proteins on adjacent filaments via the Z-disc. The proposed model is capable of explaining various SPOC phenomena based on the stochastic power-stroke mechanism of motor proteins, which responds to temporal changes in longitudinal mechanical load.

## Introduction

**Figure 1:**
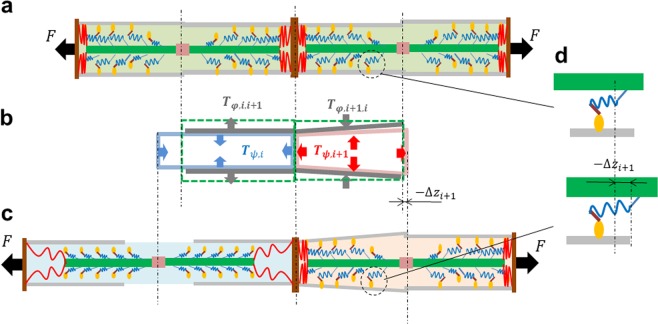
Mechanical changes in the contracted sarcomere (right) and bound actomyosin complexes inside it caused by the rapid lengthening of an adjacent sarcomere (left). Two adjacent sarcomeres were assumed to be pulled by a constant external force *F* (black arrows). The thin and thick filaments are coloured green and grey, respectively. The Z-discs are coloured brown. The red lines represent titins. Both the left and right sarcomeres were contracted in (**a**), and then the left sarcomere rapidly lengthened during relaxation, while the right sarcomere was still contracted (**c**). The mechanical interaction between the two half-sarcomeres over the central Z-disc is depicted in (**b**). From the inverse sarcomere length (SL)-lattice spacing (LS) relationship, the LS of the left half-sarcomere was reduced during lengthening (vertical blue arrows: ***T***_*ψ*,*i*_). The gap in the LS between the two half-sarcomeres produces lateral expansion (left grey arrows: ***T***_*φ*,*i*,*i*+1_) and compression (right grey arrows: ***T***_*φ*,*i*+1,*i*_) in the left and right half-sarcomeres, respectively. As a result of this transverse compressive tension (grey arrows: ***T***_*φ*,*i*+1,*i*_), the transverse expansion tension (vertical red arrows: ***T***_*ψ*,*i*+1_) originating from the inverse SL-LS relationship was generated. This was accompanied by an increase in the longitudinal tension (horizontal red arrows: ***T***_*ψ*,*i*+1_). The increase in the longitudinal load of the half-sarcomere #*i*+1 was balanced by increases in the rod strains of the bound actomyosin complexes inside it (**d**). In this way, rapid relaxation of one sarcomere (left) affects the actomyosin dynamics in the adjacent sarcomeres (right).

Myosin molecules are motor proteins that generate contractile forces in muscle. These protein motors are arranged in thick filaments, which bind with actin molecules in the thin filaments to form the actomyosin complex (Fig. 1a). The sarcomere is the basic contractile unit, in which the thick and thin filaments slide past each other during repeated shortening and lengthening. A myofibril is composed of repeating sections of sarcomeres connected in a series at the Z-discs. It is generally accepted that contractile tension is generated by structural changes in the actomyosin complex, called the power-stroke^1–3^. In the present study, we propose a mechanical transmission mechanism between adjacent sarcomeres in a single myofibril that explains the ensemble behaviour of the actomyosins, which are important for the quick relaxation of muscles.

In general, the contractile forces in sarcomeres increase when the sarcoplasmic reticulum releases Ca^2+^ ions, and decreases when Ca^2+^ ions are sequestered again in the sarcoplasmic reticulum. However, muscles can relax more rapidly than can be explained by the decay rate of free Ca^2+^ ions. For example, in a healthy beating heart, the left ventricular pressure falls much more rapidly than the free Ca^2+^ ion concentration, which is important for the quick filling of blood, especially with fast heart rates^4^. In the flapping asynchronous muscles of insect wings, the sarcomeres must relax in tandem without Ca^2+^-ion regulation. This phenomenon cannot be understood by simply considering the collective behaviour of actomyosin complexes in a single sarcomere. Rather, the interactions between actomyosin complexes of different sarcomeres must be included.

When studying the relaxation mechanism of muscles, clues are provided by the spontaneous oscillation (SPOC) process^5–7^, in which waves of rapid lengthening in sarcomeres propagate along a myofibril without Ca^2+^-ion regulation. For a single half-sarcomere model, we previously reported that this SPOC can be explained based on the stochastic power-stroke mechanism^8^. In particular, in our model, the rapid lengthening is caused by the avalanche of reversal power-strokes after a fully activated state is reached. In the present study, we examine how these avalanches propagate along each half-sarcomere in the myofibril, and can occur almost synchronously in the myofibril under some specific conditions^9,10^.

When formulating a microscale momentum equation, as with the sarcomere mechanics, the inertia term is often ignored to allow consideration of just the frictional force, together with the passive and active forces. Therefore, the mechanical equation used in this study does not have the second order time derivative. As such, it is not a type of wave propagation equation. Further, if the frictional force is negligibly small, all of the sarcomeres pull on adjacent sarcomeres with the same contractile tension, which is equal to the external tension imposed on the end of the myofibril. Thus, wave propagation cannot be explained if only the longitudinal mechanical equilibrium is considered, as the mechanical relationships between any pair of sarcomeres are the same. As a result, the equilibrium condition in the transverse direction is required to obtain wave propagation, which combines the relationships between adjacent sarcomeres. Indeed, Sato *et al*.^11^ reproduced the SPOC wave with a numerical model by connecting the sarcomeres with a lateral elasticity that acts to reduce the differences in the lattice spacing (LS) between adjacent sarcomeres. In that study, the authors also assumed an inverse relationship between sarcomere length (SL) and LS (the inverse SL-LS relationship), in which the LS decreased as the SL increased. The key component of the actomyosin dynamics that caused SPOC in their model was the dependence of the attachment rate constant on the LS.

However, we found another plausible mechanism for SPOC within a single sarcomere based on stochastic power-strokes^8^, in which the response of the actomyosin dynamics to changes in the SL is the critical factor. Thus, to reproduce the wave propagation with our stochastic actomyosin model, there must be some mechanism that transforms the decreases in the transverse widths into an increase in the longitudinal mechanical loads imposed on the protein motors (Fig. 1b,c). For simplicity, in the present study we assumed that there were only transverse isotropic deformations for each half-sarcomere, and we focused on the role of their deformation energy per unit volume in the unloaded condition, which can be described by:

1 $$\psi (\lambda ,\mu )=\frac{1}{2}{k}_{LL}{(\lambda -1)}^{2}+{k}_{TT}{(\mu -1)}^{2}+\frac{1}{2}{k}_{LT}R{(\lambda ,\mu )}^{2}.$$

Here, *λ* and *μ* are the stretching parameters in the longitudinal and transverse directions, respectively. The first and second terms are the deformation energies associated with the longitudinal and transverse elasticities of the half-sarcomere, respectively. Each term is determined based solely on the strain in its associated direction. The last term is a weak penalty term associated with the inverse SL-LS relationship, which constrains the half-sarcomere deformation to make |*R*(*λ*, *μ*)| small. The parameter *k*_*LT*_ represents the sarcomere stiffness associated with this penalty. Numerous experimental studies^12–16^ suggest that the inverse SL-LS relationship can be approximated with a linear function, as follows:

2 $$R(\lambda ,\mu )=\lambda -1+2{\beta }_{R}(\mu -1).$$

The first Piola-Kirchhoff stress tensor associated with the potential *ψ* is represented by:

3 $${{\boldsymbol{T}}}_{\psi }(\lambda ,\mu )={T}_{\psi L}(\lambda ,\mu ){{\boldsymbol{e}}}_{Z}\otimes {{\boldsymbol{e}}}_{Z}+{T}_{\psi T}(\lambda ,\mu )({{\boldsymbol{e}}}_{X}\otimes {{\boldsymbol{e}}}_{X}+{{\boldsymbol{e}}}_{Y}\otimes {{\boldsymbol{e}}}_{Y}).$$

Here, *X* and *Y* are the Lagrangian coordinates in the transverse directions, *Z* is the Lagrangian coordinate in the longitudinal direction along the unloaded myofibril, {***e***_*X*_, ***e***_*Y*_, ***e***_*Z*_} is the associated orthonormal basis, and $\otimes$ represents the tensor product. For a given unit vector, ***N*** = *N*_*X*_***e***_*X*_ + *N*_*Y*_***e***_*Y*_ + *N*_*Z*_***e***_*Z*_, ***T***_*ψ*_***N****dS* = (*T*_*ψL*_*N*_*Z*_***e***_*Z*_ + *T*_*ψT*_*N*_*X*_***e***_*X*_ + *T*_*ψT*_*N*_*Y*_***e***_*Y*_)*dS* gives the force acting on the cross sectional area *dS*, which is perpendicular to ***N***. The longitudinal and transverse components are given by:

4 $${T}_{\psi L}(\lambda ,\mu )=\frac{\partial \psi }{\partial \lambda }(\lambda ,\mu )={k}_{LL}(\lambda -1)+{k}_{LT}R(\lambda ,\mu ),$$

5 $${T}_{\psi T}(\lambda ,\mu )=\frac{1}{2}\frac{\partial \psi }{\partial \mu }(\lambda ,\mu )={k}_{TT}(\mu -1)+{\beta }_{R}{k}_{LT}R(\lambda ,\mu ).$$

Based on the definition of the first Piola-Kirchhoff stress tensor, these components represent just the tensions acting on each unit area in the unloaded half-sarcomere. The factor 1/2 is multiplied by ∂*ψ*/∂*μ* in equation (5) because the transverse space is two-dimensional. As a result, the infinitesimal energy increment per unit volume in the unloaded sarcomere is δ*ψ* = −*T*_*ψL*_*δλ* − 2*T*_*ψT*_*δμ*. Note that our model of the inverse SL-LS relationship is different from that adopted in Sato *et al*.^11^, in which the longitudinal component *k*_*LT*_*R*(*λ*, *μ*) in equation (4) is not present. This term plays an important role in converting the transverse load to the longitudinal load in our model. The direction of the stress from the inverse SL-LS relationship within ***T***_*ψ*_ is determined by the sign of *R*(*λ*, *μ*). If *R* > 0, the forces are directed inward to reduce *R*. If *R* < 0, they act outwardly to increase *R*.

The physical mechanism of the inverse SL-LS relationship remains controversial. Ertbjerg *et al*.^13^ supported the constant volume assumption in the so-called pre-rigor state, and argued that the water capacity of the sarcomere is based on various interactions between the water molecules and the sarcomere proteins, including the thin and thick filaments. Li *et al*.^16^ suggested a possible contribution from titin (Fig. 1c), and although this hypothesis did not necessarily support the constant volume assumption, the experimental results obtained for relaxed single myofibrils were consistent with volume preservation (i.e., *β*_*R*_ = 1). However, for activated muscles, Yagi *et al*.^17^ observed that sarcomere volume decreased by approximately 15% when the SL was shortened from 2.3 μm to 1.8 μm during contraction. Further, the relationships reported for the activated sarcomere by Konhilas *et al*.^15^ and for the post-rigor state by Ertbjerg *et al*.^13^ fit well with *β*_*R*_ = 2.

With respect to the elasticity with differences in LS between adjacent half-sarcomeres, we defined the following expressions for the deformation energy per unit volume in the unloaded condition:

6 $${\phi }_{i}({\mu }_{i},{\mu }_{i+1})=\{\begin{array}{cc}{k}_{MM}{({\mu }_{i}-{\mu }_{i+1})}^{2}, & i=1,3,\cdots \\ \,{k}_{MZ}{({\mu }_{i}-{\mu }_{i+1})}^{2}, & i=2,4,\cdots \end{array},$$

where *μ*_*i*_ and *μ*_*i*+1_ are the transverse stretching parameters of the left and right half-sarcomeres, respectively. The half-sarcomeres are separated by the M-band for the top expression, and are separated by the Z-disc for the lower expression. Because Telley *et al*.^18^ observed a clear difference in the transfer times of half-sarcomere lengthening across these interfaces, we applied various stiffness parameters in the present study. Using the above definition of elastic energy (equation (6)), the stress tensor act*i*ng on the *i*-th half-sarcomere caused by its interaction with the *i* + 1-th half-sarcomere is given by:

7 $${{\boldsymbol{T}}}_{\phi ,i,i+1}({\mu }_{i},{\mu }_{i+1})=\frac{1}{2}\frac{\partial {\phi }_{i}}{\partial {\mu }_{i}}({\mu }_{i},{\mu }_{i+1})({{\boldsymbol{e}}}_{X}\otimes {{\boldsymbol{e}}}_{X}+{{\boldsymbol{e}}}_{Y}\otimes {{\boldsymbol{e}}}_{Y}),$$

while the stress tensor acting on the *i* + 1-th half-sarcomere because of its interaction with the *i*-th half-sarcomere is given by:

8 $${{\boldsymbol{T}}}_{\phi ,i+1,i}({\mu }_{i},{\mu }_{i+1})=\frac{1}{2}\frac{\partial {\phi }_{i}}{\partial {\mu }_{i+1}}({\mu }_{i},{\mu }_{i+1})({{\boldsymbol{e}}}_{X}\otimes {{\boldsymbol{e}}}_{X}+{{\boldsymbol{e}}}_{Y}\otimes {{\boldsymbol{e}}}_{Y}).$$

The magnitudes of ***T***_*φ*,*i*,*i*+1_ and ***T***_*φ*,*i*+1,*i*_ are the same, but their directions are opposite. These stresses act to make the differences between the transverse stretches *μ*_*i*_ and *μ*_*i*+1_ smaller.

Using the elastic energies given in equations (1) and (6), wave propagation can be understood as follows. We assume that two sarcomeres pulled by a constant external force start in a contracted state (Fig. 1a), and suddenly one half-sarcomere (#*i*) is rapidly relaxed and lengthened (Fig. 1b). The following mechanical propagation can then be considered. First, from the inverse SL-LS relationship, the inward tension *T*_*ψT*,*i*_ develops in the lengthened half-sarcomere (#*i*) because the lengthening process increases *R*(*λ*_*i*_, *μ*_*i*_). Next, the elasticity associated with the lattice alignment tends to increase the inward transverse tension ***T***_*φ*,*i*+1,*i*_(*μ*_*i*_, *μ*_*i*+1_) in the adjacent half-sarcomere (#*i* + 1), which is still activated. Third, the inverse SL-LS relationship increases the outward longitudinal tension *T*_*ψL*,*i*+1_, extending the half-sarcomere. Fourth, this additional longitudinal force acts on the bound actomyosin complex (Fig. 1c). This increase in the contractile active force is achieved by an increase in the strain of the myosin rods (Fig. 1d). Finally, the increase in the myosin rod strain facilitates reversal power-strokes, resulting in the observed half-sarcomere lengthening.

**Figure 2:**
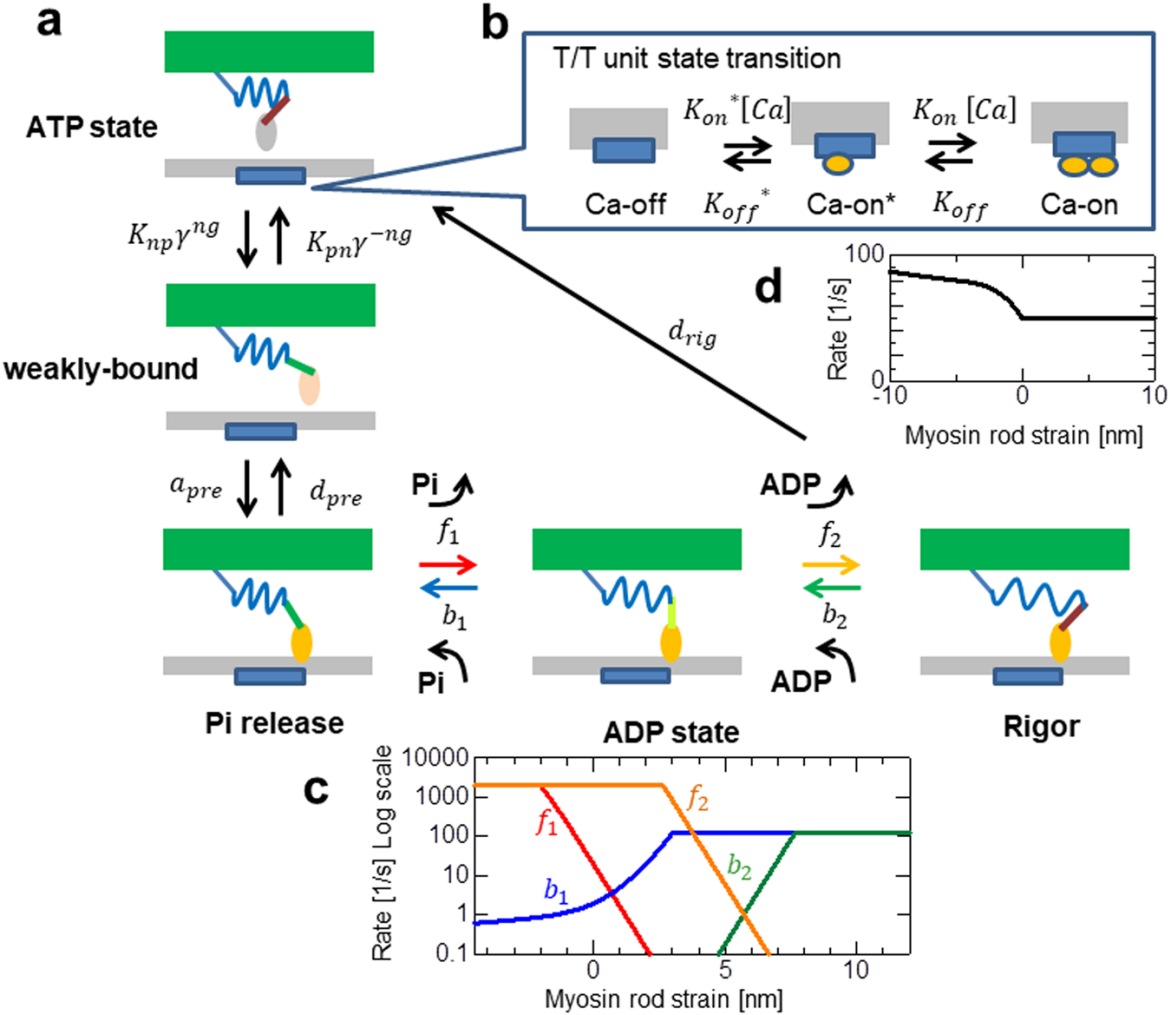
Stochastic state transition model of the actomyosin complex. The active tension was caused by three attached states (Pi-release, ADP state, and Rigor) (**a**). The transitions between the ATP and the weakly-bound states (**a**) were influenced by the state of the T/T unit (**b**) above the myosin head through the coefficients *K*_*np*_ and *K*_*pn*_, along with the states of the neighbouring myosins via the cooperative factors *γ*^*ng*^,*γ*^−*ng*^ (γ = 60). Here, *ng* = 0,1,or2 is the number of neighboring myosin heads either in the weakly-bound or the bound states. The rate constants *f*_1_, *b*_1_, *f*_2_, *b*_2_ for transitions between the bound states are given as functions of the rod strain (**c**). The rate constant for the detachment from the Rigor to ATP states is also given as a function of the rod strain (**d**). Details are given in Methods.

In the above framework, the response of the actomyosin complex to an increase in rod strain is also an important factor in mechanical propagation. In this simulation study, we applied a stochastic cross-bridge cycling model^19^, as depicted in Fig. 2. In this model, the rate constants of the power-stroke (*f*_*k*_) and the reversal power-stroke (*b*_*k*_) are determined using a function of the rod strain, such that they fulfil the relationship given by statistical equilibrium:

9 $$\frac{{f}_{k}(x)}{{b}_{k}(x+{s}_{k})}=\exp (\frac{{E}_{k-1}+{W}_{rod}(x)-{E}_{k}-{W}_{rod}(x+{s}_{k})}{{k}_{B}T}),$$

where *k*_*B*_ and *T* denote the Boltzmann constant and the temperature, respectively, and *E*_*k*−1_ and *E*_*k*_ are the free energies of the actomyosin complex before and after the power-stroke, respectively, using a stroke distance *s*_*k*_. The value *x* is the rod strain before the power-stroke, and *W*_*rod*_ is the strain energy of the myosin rod.

In our numerical model, we put *n*_*F*_ one-dimensional filament pairs in each half-sarcomere to determine the active tension at time *t* generated by the bound myosin molecules:

10 $${}^{t}{T}_{{\rm{a}}{\rm{c}}{\rm{t}}}=\frac{2}{S{A}_{0}\cdot {n}_{F}}\sum _{\beta =1}^{{n}_{F}}\sum _{\alpha =1}^{{n}_{M}}{}^{t}{\delta }_{A,\alpha ,\beta }\frac{d{W}_{rod}}{dx}({}^{t}{x}_{\alpha ,\beta }).$$

Here, ${}^{t}{\delta }_{A,\alpha ,\beta }=1$ if the myosin head is in an attached state, otherwise ${}^{t}{\delta }_{A,\alpha ,\beta }=0$. *n*_*M*_ is the number of myosin molecules arranged on a thin filament at regular intervals, while *SA*_0_ is the cross-sectional area per thin filament in an unloaded half-sarcomere. The factor of 2 comes from the fact that our one-dimensional model corresponds to one of the double spirals of actin monomers along the thin filament. The number *n*_*M*_ is determined under this assumption^19^.

As for the feedback between the sarcomeric dynamics and the actomyosin dynamics, the power-stroke and reversal power-stroke are affected by changes in the half-SL via the rod strain. The relation between the time transients of the molecular and sarcomeric variables can be expressed by

11 $${}^{t}x={}^{{t}_{A}}x+{}^{t}s-({}^{t}z-{}^{{t}_{A}}z).$$

Here, *t*_*A*_ is the most recent time at which the myosin molecule was attached, ${}^{{t}_{A}}x$ is the initial strain at the attachment, ^*t*^*s* is the total power-stroke distance after attachment, and ^*t*^*z* is the half-sarcomere shortening length from the unloaded condition:

12 $${}^{t}z=-\frac{S{L}_{0}}{2}({}^{{\rm{t}}}\lambda -1),$$

where *SL*_0_ is the unloaded SL. Thus, half-sarcomere shortening ($\dot{z} > 0$) implies a decrease in the rod strain, facilitating a power-stroke transition, which corresponds to an increase in *f*_*k*_(*x*)/*b*_*k*_(*x* + *s*_*k*_) in equation (9), while half-sarcomere lengthening ($\dot{z} < 0$) implies an increase in the rod strain, resulting in the facilitation of a reversal power-stroke (i.e., a decrease in *f*_*k*_(*x*)/*b*_*k*_(*x* + *s*_*k*_) in equation (9)). In particular, when sarcomere shortening slows down, and the high-load, greater-rod strain population increases, an avalanche of reversal power-strokes can be easily triggered by a small increase in the SL.

Note that with our numerical cross-bridge model, the sarcomeric oscillation can be reproduced even with only a single half sarcomere (Supplementary Fig. S1a,c,e; Supplementary Fig. S2a−d) if it is connected to the external spring. The non-oscillatory behaviour under the isometric condition (Supplementary Fig. S1b,d,f) indicates that this oscillation originates from the instability, given by coupling the cross-bridge dynamics with the external boundary condition that allows sarcomeric length change in response to the active tension, as analysed mathematically in our previous work^8^. We suggest that the avalanche of reversal power-strokes and its propagation along the myofibril also occurs in the natural physiological condition of the cardiac muscle, and is important for the quick relaxation during the diastolic phase in the cardiac cycle, as we previously simulated^4^. Note that the SPOC in the present study should be distinguished from the forced oscillation subject to small (0.23%) sinusoidal length change^20^. The former allows more than 3% (30 nm) length change even for the asynchronous insect flight muscle (IFM), which is much larger than the total working stroke size of 10 nm. This indicates that the sarcomeric lengthening in SPOC is induced by the increased current of detachments either from the Rigor state^11^ or from the Pi-release state (Fig. 2a) via the reversal power-strokes, but not by the population shift within the bound states.

In the present study, we analysed the roles of the inverse SL-LS relationship, the lattice space alignment, and the reaction of the actomyosin complexes (both qualitatively and quantitatively) on changes in the longitudinal mechanical load in a standard SPOC model^5–7,11^, synchronous SPOC under isotonic conditions^9^, and the high-frequency oscillation of IFMs^21^. More specifically, we estimated the magnitudes of the elastic coefficients *k*_*MZ*_, *k*_*MZ*_, and *k*_*LT*_ required to reproduce the experimental results.

## Results

### Standard SPOC

**Figure 3:**
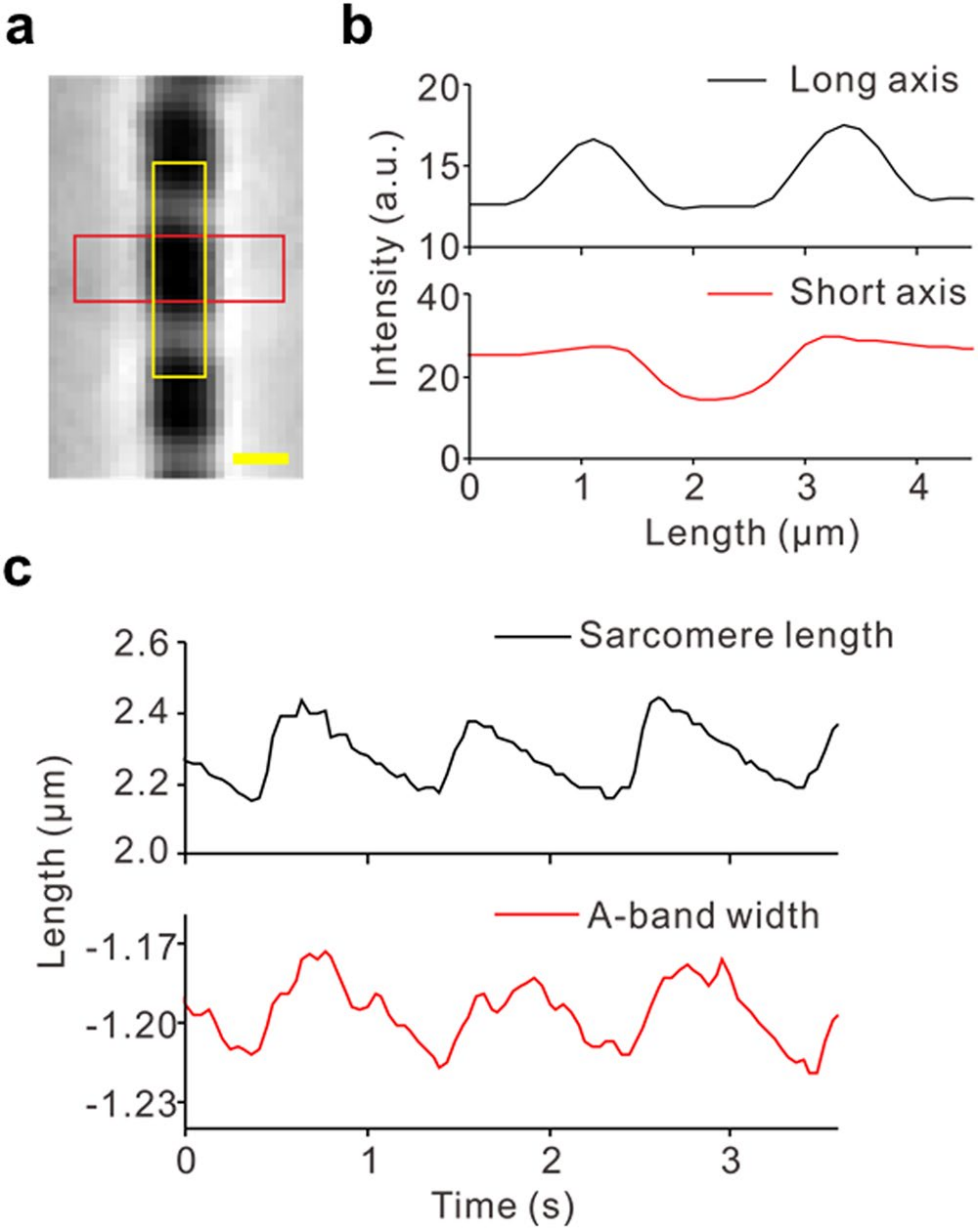
The inverse SL-LS relationship of an active sarcomere during the SPOC with a single myofibril. The myofibril was taken from a rabbit iliopsoas muscle. (**a**) A snapshot of a myofibril focused on the sarcomere, for which the SL (yellow) and the width of the A-band (red) were recorded. The scale bar shown in the lower right of (**a**) is 1 μm. (**b**) Plot of the profile of the rectangular area outlined with yellow and red in (**a)** using black and red lines, respectively. (**c**) Temporal changes in SL (black) and width of the A-band (red). The negative of the A-band width is plotted to examine the relationship with SL.

**Figure 4:**
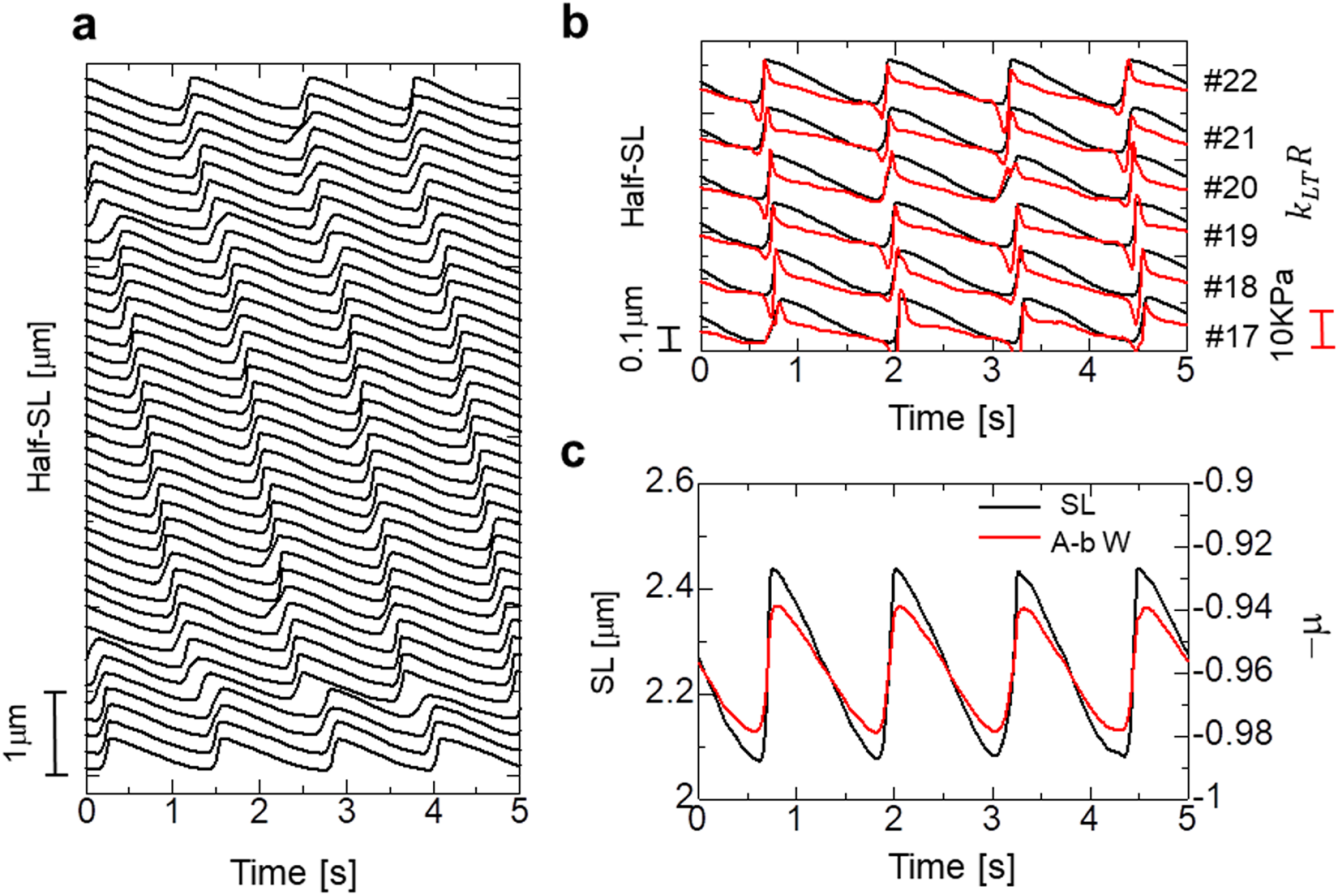
Numerical results of the standard SPOC model. The parameters for the passive properties are given as *k*_*MM*_ = 1 MPa, *k*_*MZ*_ = 0.5 MPa, *k*_*LT*_ = 0.1 MPa, *k*_*LL*_ = 0.01 MPa, and *k*_*TT*_ = 0.1 MPa. (**a**) The spatiotemporal pattern of the half-SL oscillations for the myofibril model consisting of 40 half-sarcomeres. (**b**) Time transients of the half-SL (black) and the longitudinal passive force (red: *k*_*LT*_*R*) generated by the inverse SL-LS relationship superposed for the half sarcomeres from #17 to #22. The rapid drop of *k*_*LT*_*R* before lengthening at #*i* was caused by the lengthening of #*i*+1. There were rare cases in which the lengthening started without a drop in *k*_*LT*_*R* (see #20). In these cases, the lengthening started from its own periodicity slightly before the lengthening of neighbouring half-sarcomeres. (**c**) The time transients of the SL (black) and the transverse stretching parameter μ of the sarcomere containing #19 and #20 half-sarcomeres. The negative of μis plotted to examine the relationship with SL.

Figure 3 shows our findings of an inverse SL-LS relationship during SPOC, in which the SL and the width of the A-band in the myofibril were observed in rabbit iliopsoas muscle (Fig. 3a), which were evaluated based on the intensities of successive captured images. The resulting plots of time transients (Fig. 3c) show the inverse SL-LS relationship consistent with *β*_*R*_ = 2 (Supplementary Fig. S3a). Therefore, we used *β*_*R*_ = 2 in the numerical model analysed below. A certain amount of dispersion can also be observed in the plots of the SL and A-band width (Supplementary Fig. S3a), indicating a larger stiffness for the lattice alignment (*k*_*MM*_, *k*_*MZ*_) than the stiffness for the inverse SL-LS relationship (*k*_*LT*_). Together with the transfer time of lengthening (50 ms per sarcomere) in this study, we used *k*_*MM*_ = 1 MPa, *k*_*MZ*_ = 0.5 MPa, *k*_*LT*_ = 0.1 MPa, *k*_*TT*_ = 0.1 MPa, and *k*_*LL*_ = 0.01 MPa in our numerical model with 40 half-sarcomeres, in which the standard SPOC was reproduced (Fig. 4).

The ratio of *k*_*MM*_ to *k*_*MZ*_ was determined based on the observation that the transfer time for half-sarcomere lengthening over the M-band is approximately one-half of that for over the Z-disc^18^. The spatiotemporal pattern of the half-SL in Fig. 4a fits well with our experimental results with respect to the transfer time and the time-transient wave profile. The time transients of the passive tension *k*_*LT*_*R*(*λ*, *μ*) (Fig. 4b) from the inverse SL-LS relationship are in good agreement with the picture of wave propagation depicted in Fig. 1. Namely, in many cases the sharp drops in the passive tension are seen just prior to half-sarcomere lengthening. The ratio of the amplitude of the A-band width oscillation to that of the SL oscillation was also reproduced, as shown in Fig. 4c.

### Isotonic SPOC

**Figure 5:**
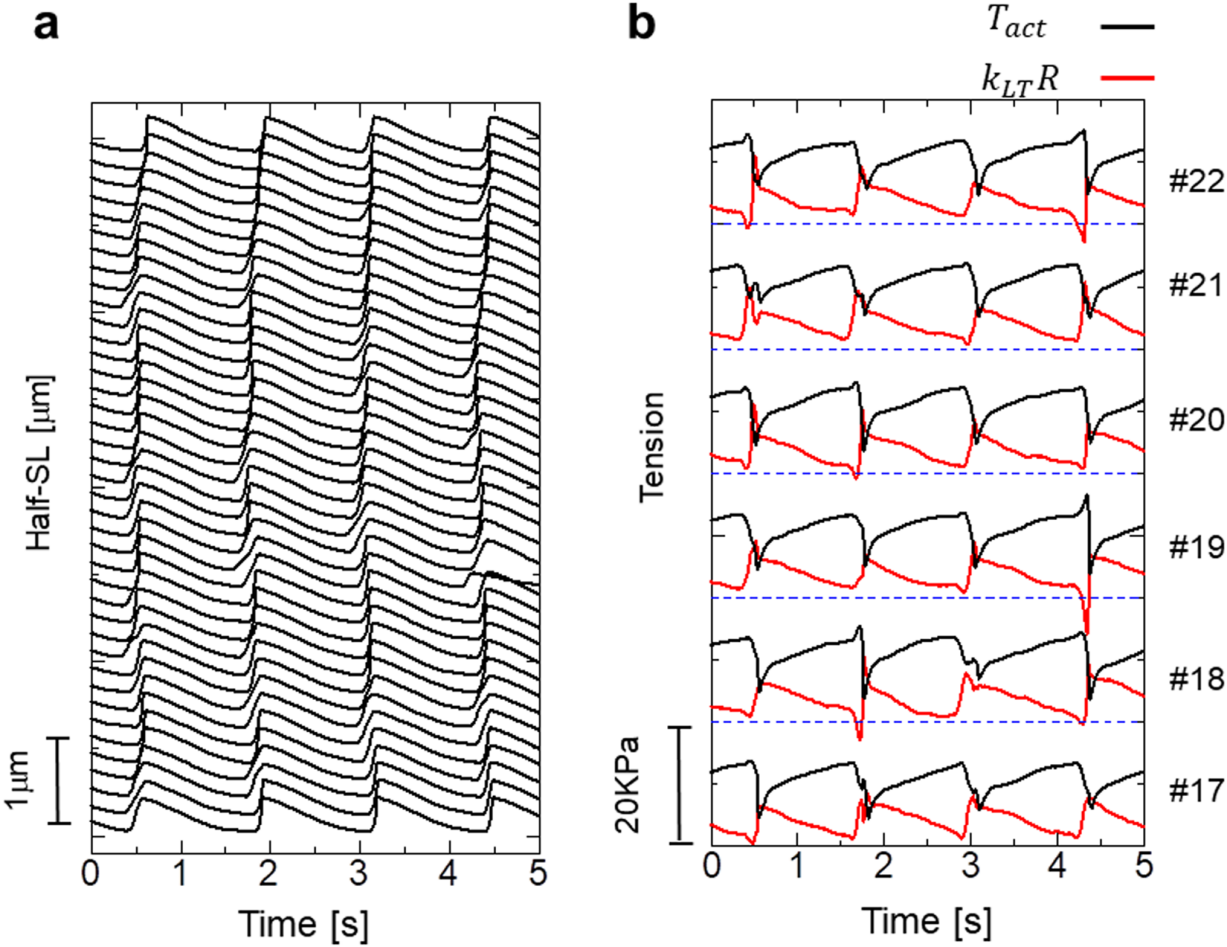
Numerical results of the SPOC model with isotonic boundary conditions. The parameters for the passive properties were the same as those applied to the standard SPOC model (*k*_*MM*_ = 1 MPa, *k*_*MZ*_ = 0.5 MPa, *k*_*LT*_ = 0.01 MPa, *k*_*LL*_ = 0.01 MPa, and *k*_*TT*_ = 0.1 MPa). The free end of the myofibril was pulled by a constant tension of 15 KPa. (**a**) The spatiotemporal pattern of Half-SL oscillations for the myofibril model consisting of 40 half-sarcomeres. (**b**) The time transients of the active tension (*T*_*act*_: black) and the longitudinal passive force (red: *k*_*LT*_*R*) generated by the inverse SL-LS relationship are superposed for the half sarcomeres from #17 to #22. The blue broken horizontal lines indicate the zero level for each half-sarcomere. These findings show that the lack of active tension was compensated for mostly by the inverse SL-LS passive force, *k*_*LT*_*R*, to maintain a constant longitudinal total tension of 15 KPa. When the half-sarcomeres had slight delays in their active tension drops compared with the neighbouring sarcomeres, the increases in the active tensions were observed just before their drops. These increases caused by drops in *k*_*LT*_*R* triggered the lengthening, and contributed to the synchronization of the lengthening process.

In Fig. 5a, the spatiotemporal pattern of the half-SL is shown in the isotonic condition, in which the right end of the previous myofibril in a standard SPOC was pulled by a constant tension of 15 KPa, rather than being connecting by an external spring. As reported experimentally by Yasuda *et al*.^9^, the synchronized oscillation was also reproduced with our numerical model. In the previous auxotonic condition, because the force pulling the myofibril changed during the lengthening of the half-sarcomeres, the active tensions fluctuated accordingly (Supplementary Fig. S5). Conversely, the active tensions were influenced only when neighbouring half-sarcomeres became lengthened, and the lack of active tension was compensated for mostly by the passive force given by the inverse SL-LS relationship in the isotonic case (Fig. 5b).

### Asynchronous insect flight muscles

Another method to enhance the synchrony of oscillation is to reduce the transfer time of the half-sarcomere lengthening process by increasing the stiffness parameters *k*_*MM*_ and *k*_*MZ*_, which are related to the lattice alignment of neighbouring half-sarcomeres. Iwamoto *et al*.^10^ found that the regularity of the lattice structure of flight muscles in bumble bees along the long-axis was not confined to within a single sarcomere, but rather extended over the entire length of the myofibril. This suggests a much stronger effective stiffness associated with the lattice alignment for the IFM than that for the cardiac muscle, which increases the synchrony of oscillation.

**Figure 6:**
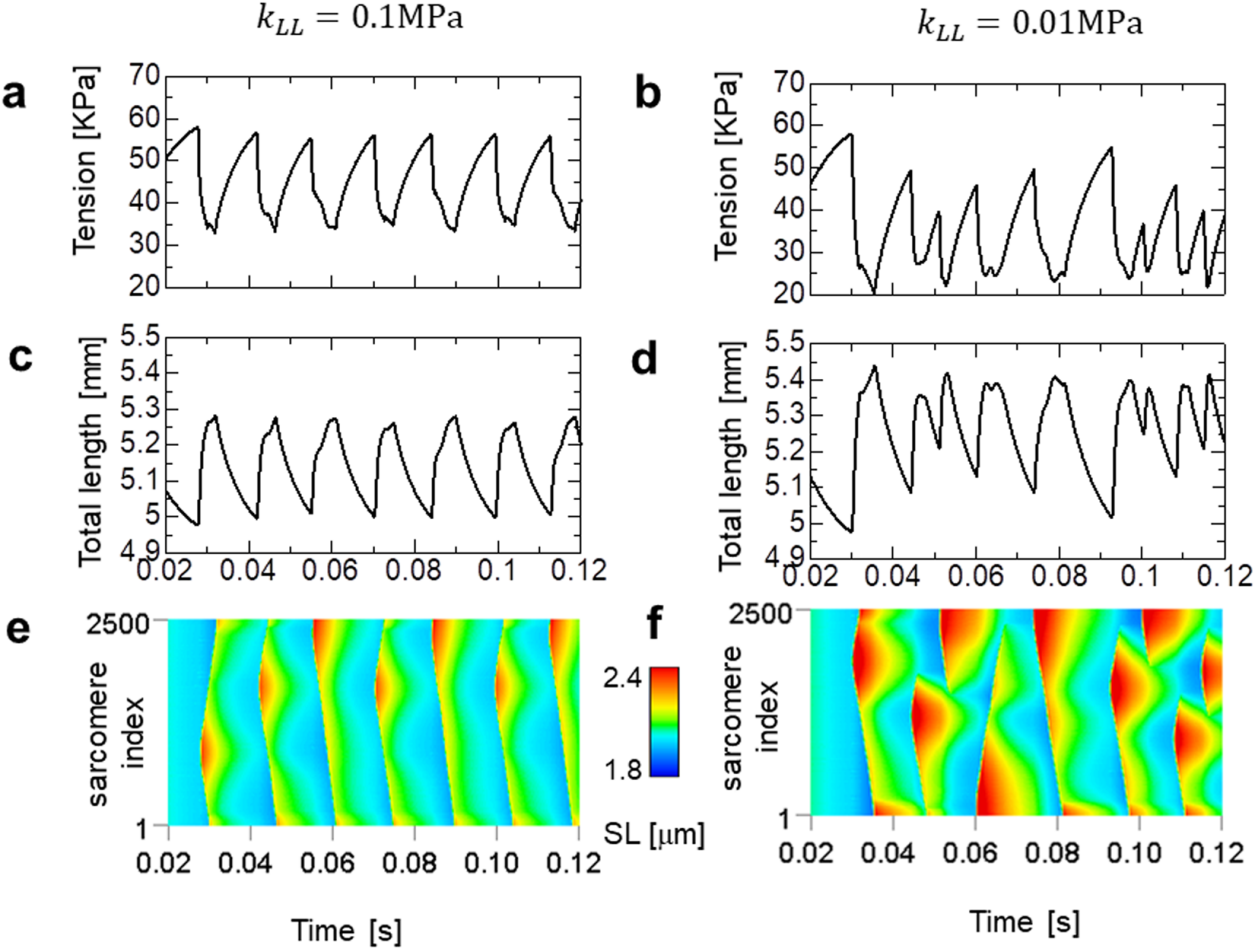
Numerical results of SPOC in the insect flight myofibril model consisting of 5,000 half-sarcomeres. The parameters of the lattice alignment are given by multiplying 50,000 to the standard SPOC parameters to enhance the traveling speed 50,000 times (*k*_*MM*_ = 50 GPa, *k*_*MZ*_ = 25 GPa). The parameter for the inverse SL-LS relationship is given by multiplying the standard SPOC parameter by 20 (*k*_*LT*_ = 0.2 MPa). Two longitudinal stiffness parameter values *k*_*LL*_ = 0.1 MPa (left [**a,c**]) and *k*_*LL*_ = 0.01 MPa (right [**b,d,f**]) are used to see the influence of this parameter. (**a,b**) The time transient of the tension generated at the free end of the myofibril, in which resistance with a friction coefficient ${\gamma }_{LE}=20{\rm{Pa}}\cdot {\rm{s}}/{\rm{mm}}$ is imposed. (**c,d**) The time transient of the total myofibril length. (**e,f**) The spatiotemporal pattern of SL for all sarcomeres.

In the present study, we attempted to reproduce the behaviour of asynchronous beetle flight muscles, which operate at a frequency of 60–80 Hz^22^, using our myofibril model consisting of 5,000 half-sarcomeres, with a total length of approximately 5 mm. The high frequency oscillations for a single half-sarcomere are easily achieved by changing the rate constant factor *R*_0_ for cross-bridge cycling (Table 1) from 1 (as with standard SPOC) to 30 for the beetle flight muscle. If lengthening wave propagation through the entire myofibril (consisting of 2,500 sarcomeres) can occur within a quarter period of a 100-Hz oscillation, the transfer time for one sarcomere must be <0.001 ms (=2.5 ms/2500). Therefore, assuming that the wave propagation speed is proportional to the magnitudes of the stiffness parameters *k*_*MM*_ and *k*_*MZ*_, we applied a multiplication factor of 50,000 to the parameters used for standard SPOC, in which the transfer time was 50 ms. Specifically, we set the stiffness parameters to *k*_*MM*_ = 50GPa and *k*_*MZ*_ = 25GPa in the numerical experiments (Fig. 6).

**Table 1:** Parameters for the actomyosin dynamics. ‘Adjusted’ indicates that they were adjusted to reproduce the phenomena. For the factor *R*_0_, the different values 1 and 30 are applied to the standard SPOC and insect flight muscle models, respectively. If different parameters were applied to the standard SPOC and insect flight muscle models, both values are shown with an (S) or (I), respectively.

Parameter	Value	Unit	Reference	Parameter	Value	Unit	Reference
**ATP hydrolysis energy**				**Sarcomere Geometry**			
*E*_ATP_	76.5	pN⋅nm	^4^	*SL*_0_	1.9	μm	^32^
*k*_*B*_ *T*	4.05	pN⋅nm	*T* = 293°C	*LM*	1.65	μm	^31^
**Stroke sizes and Free energies**				*LB*	0.16	μm	^30^
*s*_1_, *s*_2_	5.0	nm	^35^	*LA*	1.0	μm	^30^
*E*_*wb*_	*E*_ATP_	pN/nm	^19^	*SA*_0_	693	nm^2^	^11^
*E*_0_	1.05*E*_*wb*_	pN/nm	^19^	**Force regulation through T/T-unit**			
*E*_1_	0.82 *E*_ATP_	pN/nm	^19^	${K}_{{\rm{on}}}^{\ast }$	50*R*_0_	1/s	adjusted
*E*_2_	0	pN/nm	^19^	${K}_{{\rm{off}}}^{\ast }$	5*R*_0_	1/s	adjusted
**Rod strain energy** ***W***_***rod***_				*K*_on_	90*R*_0_	1/s	adjusted
*k*_*xb*_	2.0	pN/nm	^19^	*K*_off_	60 *R*_0_	1/s	adjusted
*b*_*xb*_	0.05	unitless	^19^	*K*_basic_	20	1/s	^19^
*ξ*_1_	4.35	nm	^19^	*Q*	2	unitless	^19^
**Power-stroke transitions**				*μ*	20	unitless	^19^
*h*_1_	20*R*_0_	1/s	adjusted	*γ*	60	unitless	^4^
*h*_2_	0.1*R*0	1/s	adjusted	**Sarcomere friction**			
${\bar{f}}_{1}$, $\,{\bar{f}}_{2}$	2000 *R*_0_	1/s	adjusted	*γ*_*L*_	0.01	KPa⋅s	^8^
${\bar{b}}_{1}$, $\,{\bar{b}}_{2}$(S)	125*R*_0_	1/s	adjusted	*γ*_*T*_	0.01	KPa⋅s	adjusted
${\bar{b}}_{1}$, $\,{\bar{b}}_{2}$(I)	2000 *R*_0_	1/s	adjusted	**Number of elements in a sarcomere**			
**Detachment transitions**				*n*_*M*_	38	unitless	^19^
*d*_pre_	3000*R*_0_	1/s	^19^	*n*_*T*_	32	unitless	^19^
*d*_ri *g*0_	50*R*_0_	1/s	^19^	*n*_*F*_ (S)	2048	unitless	adjusted
*c*_*for*_	100*R*_0_	1/s	adjusted	*n*_*F*_(I)	1048	unitless	adjusted

To examine the influence of the longitudinal stiffness, we varied the parameters *k*_*LL*_ = 0.1 MPa (Fig. 6a,c,e) and *k*_*LL*_ = 0.01 MPa (Fig. 6b,d,f). The former value was ten times larger than that applied for the standard SPOC. For the inverse SL-LS relationship, *k*_*LT*_ = 2 MPa was adopted, which was 20 times larger than that applied for the standard SPOC. Granzier *et al*.^23^ reported a longitudinal passive stiffness (*E*_0_) approximately 20 times larger in the IFM fibre compared with a psoas fibre. In fact, Fig. 6c−f suggests that such a strong longitudinal stiffness is necessary to avoid a so-called disrupted traveling wave^11^, which caused irregular time transients in the total strain (Fig. 6d). The range of tensions (Fig. 6a,b) was similar to experimental observations^21^, while the range of strains (Fig. 6c) was slightly larger (approximately 3%−4%) than reported values^21,22^.

**Figure 7:**
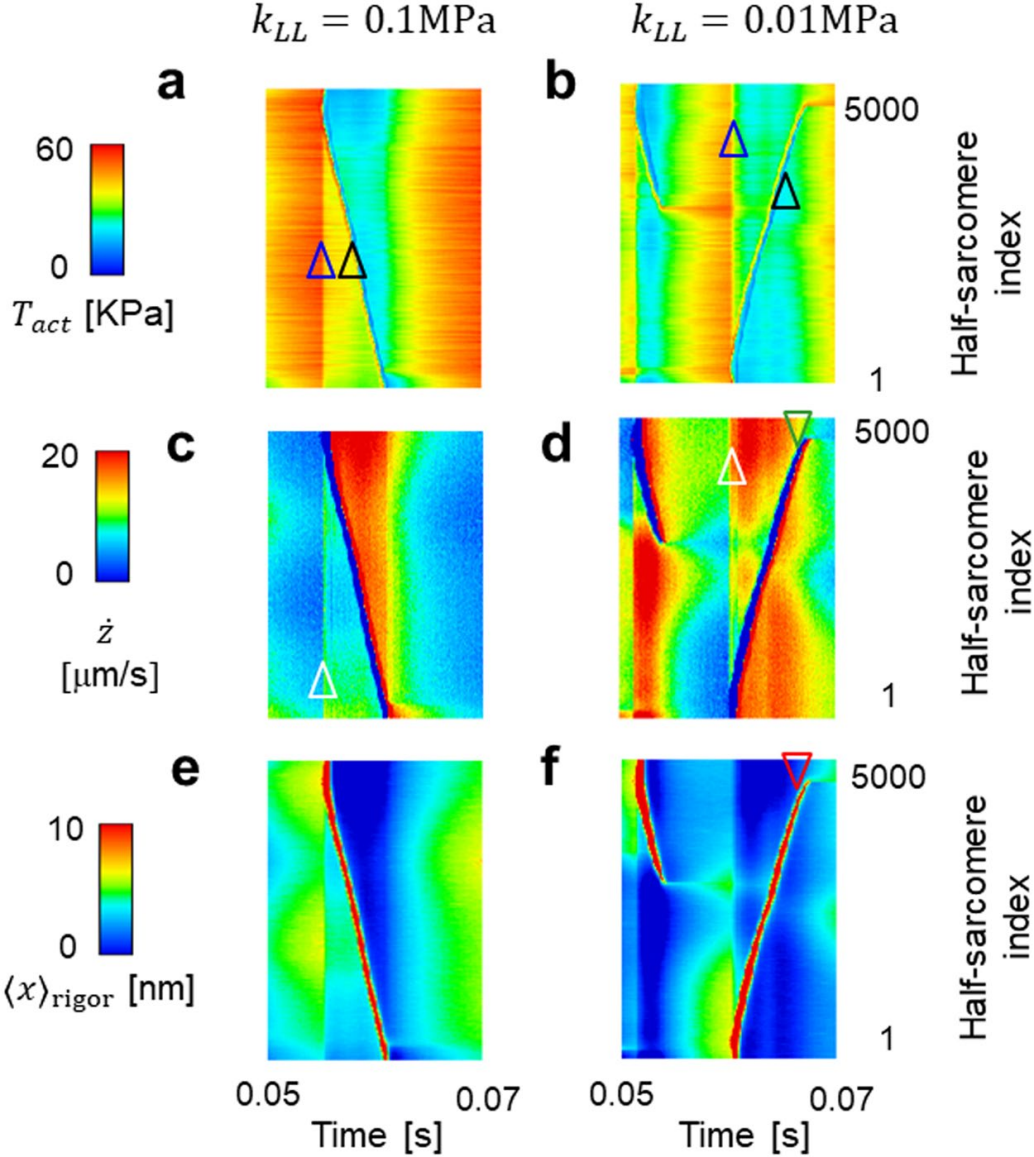
Spatiotemporal pattern of the active tension *T*_*act*_ (**a**,**b**), the half-sarcomere shortening velocity $\dot{z}$ (**c**,**d**), and the averaged rod strain in the Rigor state <*x>*_rigor_ (**e**,**f**) over the time interval (0.05 s, 0.07 s) for the insect flight myofibril model. The left side uses longitudinal stiffness *k*_*LL*_ = 0.1 MPa, while the right uses *k*_*LL*_ = 0.01 MPa. The rapid drop in the active tension was observed during lengthening (indicated by black triangles), as well as by initiation of a traveling wave (indicated by blue triangles) (**a,b**). The latter discontinuity was generated by the rapid increase in the shortening velocity (indicated by white triangles) (**c,d**). The increase in the shortening velocity is much higher in the case of *k*_*LL*_ = 0.01 MPa compared with the case of *k*_*LL*_ = 0.1 MPa. In the latter case, the shortening velocity remains large when the wave arrives (green triangle) and travel is stopped (**d**). From the large shortening velocity, the averaged rod strain in the Rigor state <*x>*_rigor_ is <0 where the traveling wave stopped (pointed by the red triangle) (**f**).

**Figure 8:**
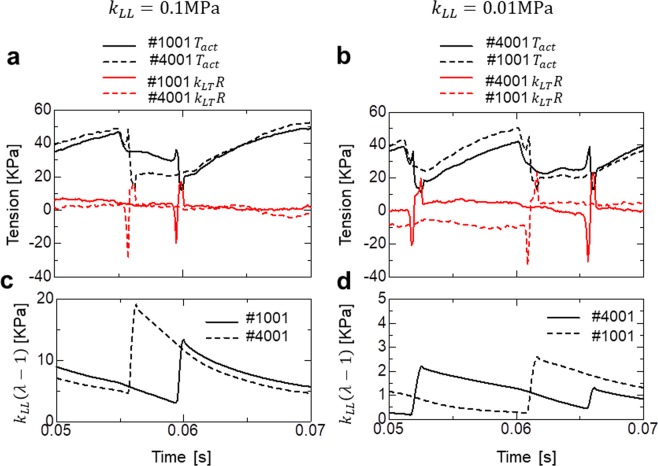
Comparison of the longitudinal mechanical equilibria during the wave traveling phase (0.05 s, 0.07 s) in the insect flight muscle myofibril model with high (left: *k*_*LL*_ = 0.1 MPa) and low (left: *k*_*LL*_ = 0.01 MPa) longitudinal elastic stiffness. The active tension *T*_*act*_ and the passive tension *k*_*LT*_*R* generated by the inverse SL-LS relationship (**a**,**b**), and the passive tension from the longitudinal elasticity *k*_*LL*_(*λ* − 1) (**c**,**d**), in the half-sarcomeres #1001 and #4001 are plotted. In the case of *k*_*LL*_ = 0.1 MPa, the active tension difference was compensated for by differences in *k*_*LL*_(*λ* − 1), while in the case of *k*_*LL*_ = 0.01 MPa, it was compensated for by differences in *k*_*LT*_*R*.

Figure 7 shows the time transients of the active tension *T*_*act*_ (Fig. 7a,b), the half-sarcomere shortening velocity $\dot{z}$ (Fig. 7c,d; equation (12)), and the averaged rod strain *x* in the ‘Rigor’ state (Fig. 7e,f; Fig. 2) for the 5,000 half-sarcomeres over the time period (0.05 s, 0.07 s). The active tension values dropped simultaneously when half-sarcomere lengthening was initiated at a particular location on the myofibril (Fig. 7a,b). The magnitude of the drop was greater when the passive longitudinal stiffness was decreased from *k*_*LL*_ = 0.1 MPa to *k*_*LL*_ = 0.01 MPa. Figure 7c,d indicates that these drops were primarily caused by a simultaneous increase in the shortening velocity. In the case of weaker longitudinal stiffness (*k*_*LL*_ = 0.01 MPa), because the high shortening velocity lasted until the lengthening wave arrived (Fig. 7d), the propagation was held back by the low average rod strain (Fig. 7f). In this case, the stimulation of neighbouring half-sarcomeres was insufficient to trigger an avalanche of reversal power-strokes. Because the total tension in all of the half-sarcomeres was equal to the tension imposed at the end of the myofibril (Fig. 6a,b), the differences in the active tensions during the propagation of the lengthening wave between the half-sarcomeres (Fig. 7a,b) was compensated for by passive tensions. This compensation was accomplished by the passive longitudinal tension *k*_*LL*_(*λ* − 1) in the case of *k*_*LL*_ = 0.1 MPa (Fig. 8a,c), and by the tension from the inverse SL-LS relationship (Fig. 8b,d) in the case of *k*_*LL*_ = 0.01 MPa. This case required much more drastic sarcomeric length changes (Fig. 6a,b; Fig. 7c,d) than the former case. Thus, a disrupted traveling wave was easily formed.

**Figure 9:**
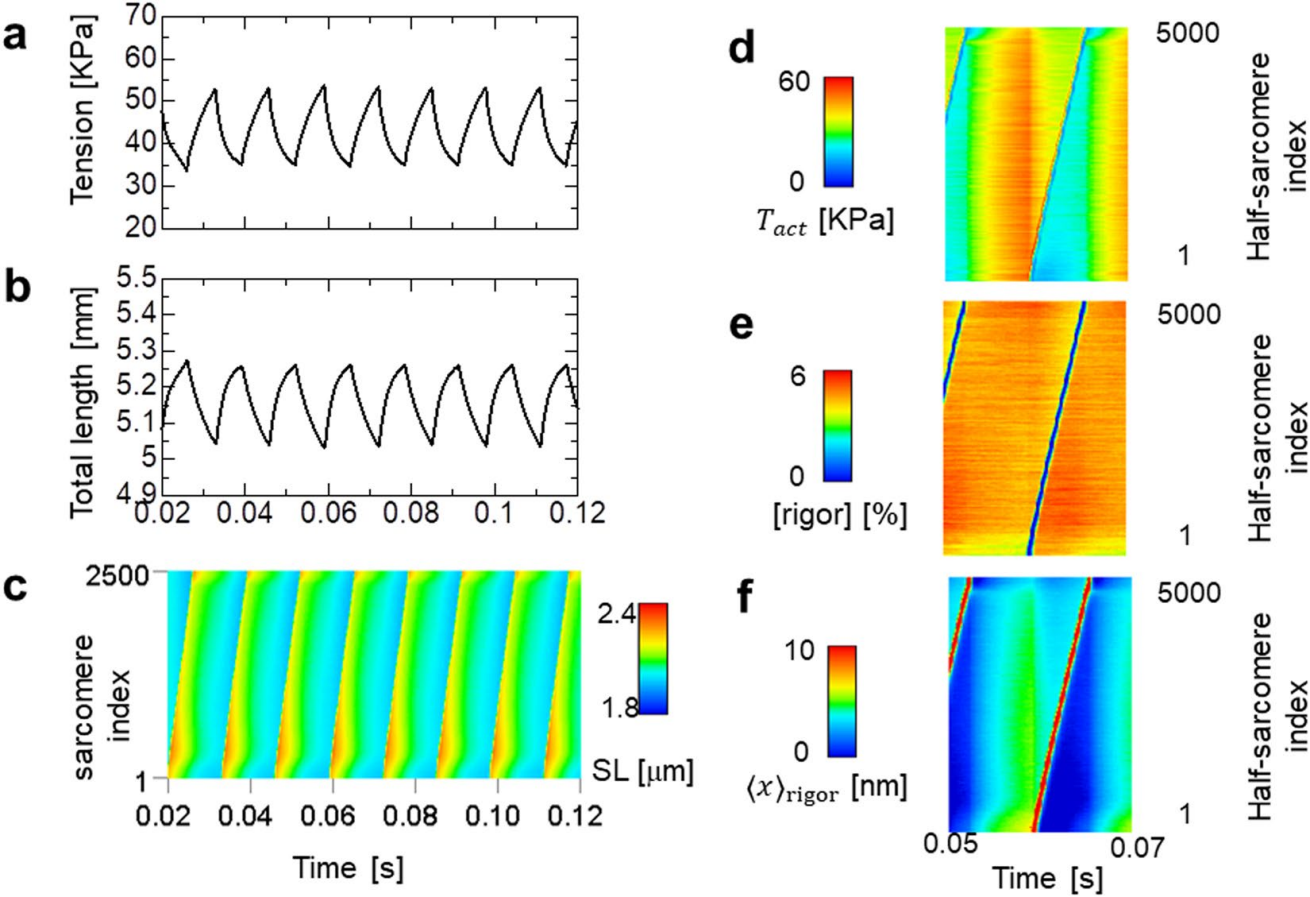
Numerical results of SPOC in the insect flight muscle myofibril model with asymmetrically constrained boundary conditions, in which the stronger transverse stiffness parameter *k*_*TT*_ was imposed on the first 250 out of 2,500 sarcomeres. The time transient of the tension generated at the free end of the myofibril (**a**) and the total myofibril length showed periodic patterns, which were different from those obtained under symmetric boundary conditions (Fig. 6). The lengthening was initiated near the boundary, where the stiffer transverse constraint was imposed (**c**). Before the initiation of lengthening, there was no clear difference in the active tension (**d**). However, the population myosin molecules in the Rigor state was smaller near the stiffer end compared with the remaining regions (**e**). To obtain the longitudinal mechanical equilibrium, this unbalance in the population was compensated for by the distribution of rod strains, as shown in (**f**), in which the average rod strain in the Rigor state <*x*>_rigor_ along the myofibril is depicted.

The spatiotemporal pattern in Fig. 6e shows alternate iterations of two patterns of wave propagation. The difference between these patterns appears in the time transients of the total strain in Fig. 6c. However, such an alternate pattern was not observed in actual IFMs^21^. Thus, it was unclear how this unique propagation pattern could be maintained in IFMs. A possible solution may relate to the difference in the boundary conditions for the transverse constraints between the two ends of the myofibril. Indeed, the difference in the thickness of the two ends of the muscular bundles, as shown in Sato *et al*.^24^, suggests a larger transverse stiffness at the thinner end compared with the thicker end, because of the transverse constraints between neighbouring myofibrils. Figure 9 shows the results in such a situation, in which the transverse stiffness *k*_*TT*_ in equation (1) linearly decreased from 1 MPa to 0.1 MPa over the first 250 sarcomeres (out of a total of 2,500 sarcomeres). In this case, the lengthening was always initiated at the stiffer transverse end (Fig. 9c). Thus, iterations of the unique profile were reproduced for the myofibril stress (Fig. 9a) and strain (Fig. 9b).

A smaller population in the Rigor state for the half-sarcomeres near the stiffer boundary compared with the other half-sarcomeres was observed (Fig. 9e) before the initiation of lengthening, while there were no clear differences in the active tension (Fig. 9d). The differences in the population were compensated for by the averaged load of the myosin molecules (Fig. 9f). Therefore, the avalanche of reversal power-strokes was triggered near the stiffer end, where the averaged rod strain in the Rigor state was higher than that in the other regions. The differences in the population were caused by a more rapid shortening following the lengthening process. This originated from the stronger transverse expansion force −*k*_*TT*_(*μ* − 1), which facilitates sarcomere shortening via the inverse SL-LS relationship.

## Discussion

For standard SPOC, the material parameters associated with the passive elastic properties in the numerical model were determined by considering the amplitudes of SL and LS, along with the transfer times in the experimental results. Although these terms were reproduced by the numerical model, there was still disagreement between the experimental and numerical results concerning the speed of the decrease in LS, compared with rising SL. Namely, while the slope of the rise in the (minus) A-band width was less than that of the SL in the experimental results (Fig. 3c), they were similar in the numerical calculations (Fig. 4c). This difference is more clearly recognized by comparing the dispersion of the SL-LS relationship (Supplementary Fig. S3a,b).

A possible explanation may be that the stiffness of the SL-LS relationship (*k*_*LT*_) was set incorrectly with respect to the stiffness of the lattice space alignment (*k*_*MM*_, *k*_*MZ*_). In this case, the distribution of the SL-LS plots can be made more dispersed by using a smaller *k*_*LT*_ value (Supplementary Fig. S4c,d). However, such a parameter change leads to difficulty in reproducing the regular traveling waves. When using *k*_*LT*_ = 0.025 MPa and *k*_*TT*_ = 0.01 MPa, in which the former was one-quarter and the latter was one-tenth of the values in the standard model, more than 50 s was required for the system to settle into a traveling wave pattern (Supplementary Fig. S4a), while only 10 s was required for the standard model (Supplementary Fig. S4b).

Based on elucidation of the wave propagation mechanism in the standard SPOC, we considered the necessary conditions required for the high-frequency oscillations in IFM consisting of a realistically large number of sarcomeres. We hypothesized that the oscillations in IFM can be determined by simply increasing the speed of the traveling waves in a standard SPOC model. By comparing the transfer times required for IFM (0.001 ms) and standard (50 ms) SPOC, a very large stiffness (*k*_*MM*_ and *k*_*MZ*_ are 50,000 times larger than those adopted in the standard SPOC model) for the lattice alignment was necessary to reproduce the physiologically-appropriate oscillations in the range of stresses and strains of the myofibril at frequencies >60 Hz. In fact, if we reduced the ratio to 25,000:1, the irregularity of the time transients became more prominent (Supplementary Fig. S6a,c) because of the slower propagation speed (Supplementary Fig. S6e). However, regarding the stiffness for the inverse SL-LS relationship (*k*_*LT*_), a stiffness at most 20 times larger was sufficient. A decrease in this parameter compared with the value previously used caused a slower propagation (Supplementary Fig. S6f), resulting in worse oscillatory behaviour (Supplementary Fig. S6b,d).

As previously described, such a high stiffness for the transverse lattice alignment agrees with the findings of Iwamoto *et al*.^10^, who concluded that the lattice order was extremely well maintained over a distance of millimetres based on an X-ray microdiffraction study of IFM. However, the molecular mechanism underlying such an extreme rigidity for lattice alignment, while still allowing for the moderate stiffness for longitudinal length changes, remains unclear. Of interest, there are specific proteins found only in IFM, including kettin, projectin, and flightin^25^, which connect the thin and thick filaments, although the known roles of these proteins are primarily for longitudinal elasticity. Future studies are required to determine whether these proteins are responsible for the extremely strong alignment.

As described in Fig. 1, sarcomere lengthening propagates step by step in the standard model of SPOC. The sarcomere lengthening for one half-sarcomere provides the drop in the passive tension from the inverse SL-LS relationship at the adjacent downstream half-sarcomere, which also induces lengthening (Supplementary Fig. S7a). However, in the case of IFM, which has a much higher stiffness for the lattice alignment, the drop in the passive tension from the inverse SL-LS relationship was not caused only by the lengthening of a single adjacent upstream half-sarcomere, but also involved multiple lengthening’s of a certain set of neighbouring upstream half-sarcomeres (Supplementary Fig. S7b). For this reason, we did not need to increase the stiffness in the inverse SL-LS relationship (×20), or the rate constants of actomyosin cross-bridge cycling (×30) from those of the standard SPOC model, as the increase of the stiffness in the lattice alignment (×50,000) to achieve the very short transfer times in the IFM oscillation.

## Methods

All the parameter values used in the numerical model described in the Methods section are listed in Table 1.

### Experimental protocol for the standard SPOC experiment

Preparation of myofibrils from rabbit psoas muscle was approved by the Institutional Animal Care and Use Ethical Committee of Waseda University, and was conducted according to Waseda University animal experiment rules. Experiments using the myofibrils were approved by the Institutional Animal Care and Use Ethical Committee of Waseda University and the University of Tokyo.

Myofibrils were obtained from the intestinal psoas muscle of white rabbits decapitated after anaesthesia by injecting 25 mg/kg sodium pentobarbital sodium into the ear vein. The intestinal psoas muscle was split into a thin bundle and immersed in a solution of 50% (vol/vol) glycerol, 0.5 mM NaHCO_3_, and 5 mM EGTA (pH 7.6) at −20 °C. Myofibrils were obtained by homogenizing the glycerinated muscle fibres in a solution containing 1 mM EGTA, 60 mM KCl, 5 mM MgCl, and 10 mM Tris-maleate buffer (pH 7.5).

SPOC induction of the myofibrils was performed by dropping a solution containing myofibrils onto a slide glass, and replacing the solution with an SPOC-inducing solution (2.2 mM ATP, 16.4 mM ADP, 10 mM MOPS, 2 mM EGTA, 14.2 mM MgCl_2_, 10 mM Pi, 41 mM KCl, pH 7.0) at a temperature of 25 °C. To obtain the SL, the peak position of the intensity profile along the myofibril was determined by fitting the intensity of the I-band to a parabolic function, and the SL was measured as the distance between consecutive peaks^26,27^. The width of the A-band was determined by the following method. Deviations of the intensity profile along the radial direction of the A-band were calculated. The peak position of each deviation was determined by fitting the intensity at the edge of the A-band to a parabolic function, and the width was measured as the distance between consecutive peaks. When this analysis was performed on immobile myofibrils in Ca^2+^-free homogenizing solution to confirm the accuracy, the coefficient of variation (CV) of the value of the width of the A-band was 0.4%, and the CV of the value of the SL was 0.7%. These CVs were relatively small compared with the amplitudes in Fig. 3c.

### Modelling of the force generating process

The rate constants for the power-stroke and the reversal power-stroke were determined based on the temporary rates ${\hat{f}}_{i}$ and ${\hat{b}}_{i}$:

$${\hat{f}}_{i}(x)={h}_{i}\exp (\frac{{E}_{i-1}+{W}_{rod}(x)-{E}_{i}-{W}_{rod}(x+{s}_{i}/2)}{{k}_{B}T}),$$

$${\hat{b}}_{i}(x+{s}_{i})={h}_{i}\exp (\frac{{W}_{rod}(x+{s}_{i})-{W}_{rod}(x+{s}_{i}/2)}{{k}_{B}T}).$$

Here, *E*_*i*−1_ and *E*_*i*_ are the free energies of the myosin head before and after the power-stroke transition, respectively. *W*_*rod*_(*x* + *s*_*i*_/2) was introduced to represent the energy barrier between the two states^8^. The contribution of the free energy of the myosin head at the barrier between the before and after states of the power-stroke is included in the constant *h*_*i*_. As shown in Fig. 2a, the power-stroke transitions are thought to accompany a release of Pi and ADP from the nucleotide binding pocket in the myosin head during the first and second power-stroke, respectively^28^. In this situation, the reversal power-stroke transitions must accompany a rebinding of these molecules. Therefore, it is reasonable to suppose that the transition rates are limited by the rates of these chemical reactions. We assumed that the rates of the chemical reactions for the release and the rebinding are given by ${\bar{f}}_{i}$ and ${\bar{b}}_{i}$, respectively. With these upper limits, the temporary rate constants were modified, as follows (Fig. 2c):

$${f}_{i}(x)=\{\begin{array}{cc}{\bar{f}}_{i}, & \,x\le {\bar{x}}_{f,i}\\ {\hat{f}}_{i}(x),\,{\bar{x}}_{f,i} <  & x\le \,{\bar{x}}_{b,i}\\ \frac{{\hat{f}}_{i}(x){\bar{b}}_{i}}{{\hat{b}}_{i}(x+{s}_{i})},\, & x > {\bar{x}}_{b,i}\end{array},$$

$${b}_{i}(x+{s}_{i})=\{\begin{array}{cc}\frac{{\hat{b}}_{i}(x+{s}_{i}){\bar{f}}_{i}}{{\hat{f}}_{i}(x)},\, & x\le {\bar{x}}_{f,i}\\ {\hat{b}}_{i}(x+{s}_{i}),\,{\bar{x}}_{f,i} <  & x\le \,{\bar{x}}_{b,i}\\ {\bar{b}}_{i}, & \,x > {\bar{x}}_{b,i}\end{array}.$$

Here, ${\bar{x}}_{f,i}$ and ${\bar{x}}_{b,i}$ are the strains at which the temporary rates reach the upper limits (${\hat{f}}_{i}({\bar{x}}_{f,i})={\bar{f}}_{i},{\hat{b}}_{i}({\bar{x}}_{b,i}+{s}_{i})={\bar{b}}_{i}$). As reported by Ishiwata *et al*.^29^, the SPOC state can be achieved in the limited range of the concentrations of Pi and MgADP for a fixed concentration of free Ca^2+^ and MgATP. For example, when we applied half of the values of the standard SPOC model to the upper limits of the reversal power-stroke rate constants (${\bar{b}}_{1}$, ${\bar{b}}_{2}$), assuming the lower concentrations of Pi and MgADP, then the SPOC state was lost (Supplementary Fig. S8). These findings suggest the importance of developing a numerical model that more precisely accounts for the effect of biochemical transitions when defining the rate constants of the power-strokes and the reversal power-strokes. This forms the basis of ongoing studies in our laboratory.

The elastic force of a myosin rod is nonlinear with respect to the strain, as reported by Kaya *et al*.^1^. We assumed that the myosin rods behaved as linear springs for positive stretches, whereas nonlinear behaviour was introduced for negative stretches because of the slack induced along the myosin rod. The strain energy *W*_*rod*_ was found by integrating the force *F*_*rod*_ from *x* = 0 defined by:

$${F}_{rod}(x)=\{\begin{array}{cc}{b}_{xb}{k}_{xb}(x+{\xi }_{1})-{F}_{1}, & \,x < -\,{\xi }_{1}\,\\ \frac{{k}_{xb}}{{a}_{xb}}(\exp ({a}_{xb}x)-1), & -{\xi }_{1}\le \,x < 0\,\\ {k}_{xb}x,\, & x\ge 0\end{array},$$

where *a*_*xb*_ and *F*_1_ are determined from the other parameters, so that the function *F*_*rod*_ and its first derivative are continuous at *ξ* = 0 and −*ξ*_1_:

$$\{\begin{array}{ccc}{a}_{xb} & = & -\frac{({\rm{l}}{\rm{n}}\,{b}_{xb})}{{\xi }_{1}}\,\\ {F}_{1} & = & \frac{{k}_{xb}(1-\exp \,(\,-\,{a}_{xb}{\xi }_{1}))}{{a}_{xb}}\end{array}.$$

### Control model of attachment and detachment

In our model, we assumed that the attachment, which represents the transition from the weakly-bound state to the Pi-release state (Fig. 2a), was allowed only in the single overlap region of the thin and thick filaments. We also assumed that the myosin molecules were arranged on a thick filament at regular intervals, except for the bare zone (B-zone). Therefore, the myosin head (#α) was situated in the single overlapping region only if the following condition was fulfilled:

$${\rm{\max }}(LA-HSL,HSL-LA)\le \frac{LB}{2}+\frac{2(\alpha -0.5)}{{n}_{M}(LM-LB)}\le HSL.$$

Here, the middle term is the distance from the centre of the sarcomere. *LM*, *LB*, and *LA* represent the lengths of the thick filament, the B-zone, and the thin filament, respectively. $HSL=\lambda \cdot S{L}_{0}/2$ is the half-SL for the stretching parameter *λ*. The parameters for the sarcomere geometry were determined from cardiac sarcomeres^30–33^.

The thin filament was divided into *n*_*T*_segments, termed troponin/tropomyosin (T/T) units (Fig. 2b). Three states (Ca-off, Ca-on*, and Ca-on) were assumed by each T/T unit. The transitions between the states of the T/T unit were determined by the Ca^2+^ concentration [Ca] and the four parameters ${K}_{{\rm{on}}}^{\ast }$, *K*_on_, ${K}_{{\rm{off}}}^{\ast }$, and *K*_off_, as shown in Fig. 2b. The transitions between the ATP and weakly-bound states (Fig. 2a) were affected by the status of the T/T unit above it, via modifications of *K*_np_ and *K*_pn_, as well as by the states of the neighbouring myosin heads through the integer *ng*. The value of *ng* (=0, 1, or 2) represents the number of neighbouring myosin heads in the weakly-bound state or the three bound states. The corresponding T/T unit index τ for the α-th myosin head is given by:

$$\tau =\,{\rm{i}}{\rm{n}}{\rm{t}}\,(\frac{z+0.5LB+(\alpha -0.5){S}_{M}+-(0.5S{L}_{0}-LA)}{{S}_{T}}).$$

Here, *S*_*M*_ = 0.5(*LM* − *LB*)/*n*_*M*_ represents the spacing of the myosin heads, and *S*_*T*_ = *LA*/*n*_*T*_ is the spacing of the T/T units. The corresponding T/T unit exists only if 1 ≤ τ ≤ *n*_*T*_. Based on this correspondence, the factors *K*_np_ and *K*_pn_ of the rate constants were given by:

$$\begin{array}{lll}{K}_{{\rm{n}}{\rm{p}}} & = & \{\begin{array}{l}{\delta }_{OV}{K}_{{\rm{n}}{\rm{p}}1}\,{\rm{i}}{\rm{f}}\,{\rm{t}}{\rm{h}}{\rm{e}}\,{\rm{T}}/{\rm{T}}\,{\rm{u}}{\rm{n}}{\rm{i}}{\rm{t}}\,{\rm{a}}{\rm{b}}{\rm{o}}{\rm{v}}{\rm{e}}\,{\rm{i}}{\rm{s}}\,{\rm{i}}{\rm{n}}\,{\rm{t}}{\rm{h}}{\rm{e}}\,{\rm{C}}{\rm{a}}-{\rm{o}}{\rm{n}}\,{\rm{s}}{\rm{t}}{\rm{a}}{\rm{t}}{\rm{e}},\\ {\delta }_{OV}{K}_{{\rm{n}}{\rm{p}}0}\,{\rm{o}}{\rm{t}}{\rm{h}}{\rm{e}}{\rm{r}}{\rm{w}}{\rm{i}}{\rm{s}}{\rm{e}}.\,\end{array}\\ {K}_{{\rm{p}}{\rm{n}}} & = & \{\begin{array}{l}{K}_{{\rm{p}}{\rm{n}}1}\,{\rm{i}}{\rm{f}}\,{\rm{t}}{\rm{h}}{\rm{e}}\,{\rm{T}}/{\rm{T}}\,{\rm{u}}{\rm{n}}{\rm{i}}{\rm{t}}\,{\rm{a}}{\rm{b}}{\rm{o}}{\rm{v}}{\rm{e}}\,{\rm{i}}{\rm{s}}\,{\rm{i}}{\rm{n}}\,{\rm{t}}{\rm{h}}{\rm{e}}\,{\rm{C}}{\rm{a}}-{\rm{o}}{\rm{n}}\,{\rm{s}}{\rm{t}}{\rm{a}}{\rm{t}}{\rm{e}},\\ {K}_{{\rm{p}}{\rm{n}}0}\,{\rm{o}}{\rm{t}}{\rm{h}}{\rm{e}}{\rm{r}}{\rm{w}}{\rm{i}}{\rm{s}}{\rm{e}}.\,\end{array}\end{array}$$

Here, *δ*_*OV*_ = 1 if the myosin head was located at the single overlapping region with the thin filament, otherwise *δ*_*OV*_ = 0. The factors *γ*^*ng*^ and *γ*^−*ng*^ (*γ* = 60) represent the nearest-neighbour cooperativity of the myosin heads, as reported by Rice^34^, which plays an important role for the force-pCa relationship^4^. We assumed that one thin filament in the three-dimensional arrangement corresponds to two thin filaments in our half-sarcomere model. This is because we assumed that cooperative behaviour exists along the tropomyosin molecules wrapped around the thin filament in a double spiral fashion, and one spiral was modelled in our half-sarcomere. The constants *K*_np0_, *K*_np1_, *K*_pn0_, and *K*_pn1_ were determined from *Q*, *K*_basic_, and *μ*, as follows:

$${K}_{{\rm{np0}}}={F}_{K}(SL)\frac{Q{K}_{{\rm{basic}}}}{\mu },\,{K}_{{\rm{np1}}}={F}_{K}(SL)Q{K}_{{\rm{basic}}},\,{K}_{{\rm{pn0}}}={K}_{{\rm{pn1}}}={K}_{{\rm{basic}}}{\gamma }^{2}.$$

Here, *μ* > 1 controls the degree of cross-bridge inhibition for the T/T units in states other than Ca-on, and *Q* controls the ratio of binding states for the myosin heads. The greater the value of *Q*, the larger the ratio of the binding states for a given Ca^2+^ concentration. To reproduce the SL (*SL* = *SL*_0_*λ*) dependence in the active contraction tension, the following function *F*_*K*_(*SL*) was multiplied to define *K*_np0_ and *K*_np1_.

$${F}_{K}(SL)=\{\begin{array}{cc}1, & SL\ge S{L}_{Q},\\ 1-{\alpha }_{Q}(S{L}_{Q}-SL), & SL < S{L}_{Q}\end{array}.$$

The values *α*_*Q*_ = 1[1/μm] and *SL*_*Q*_ = 2.2 μm were used in this study. For [Ca] (Fig. 2b), 0.2 μM and 2 μM were adopted for the standard SPOC and for the insect-flight myofibril models, respectively.

The rate constants of attachment *a*_*pre*_ and detachment *d*_*pre*_ were found based on the assumed free energies *E*_*wb*_ and *E*_0_ of the weakly-bound state and the Pi-release state, respectively:

$${a}_{pre}={d}_{pre}\exp (-\frac{{E}_{0}-{E}_{wb}}{{k}_{B}T}).$$

The initial rod strain during attachment was given stochastically based on a Boltzmann distribution determined from the rod strain energy *W*_*rod*_(*x*)^19^. The detachment rate-constant for the transition from the Rigor state to the ATP state was assumed to be strain-dependent for negative strains, as follows (Fig. 2d):

$${d}_{rig}(x)=\{\begin{array}{cc}{d}_{rig0}\exp (\,-\,{F}_{rod}(x)/{F}_{d}),\, & x < 0\\ {d}_{rig0}, & \,x\ge 0\end{array}.$$

A value of *F*_*d*_ = 6 pN was used in this study. We also considered forced detachment caused by the extreme strain of a myosin rod using the rate constant:

$${d}_{for}(x)=\{\begin{array}{cc}0, & x < {x}_{for}\,\\ {c}_{for}(\exp ({a}_{for}(x-{x}_{for}))-1), & x\ge {x}_{for}\end{array}.$$

The values of *a*_*for*_ = 0.025 [1/nm] and *x*_*for*_ = 10 nm were adopted for this study. *c*_*for*_ is listed in Table 1. Here, we assumed that forced detachment from the Pi-release state led to the weakly-bound state, while forced detachment from the ADP or the Rigor states led to the ATP state.

### Simulation protocol

Within each half-sarcomere, the following contractile tensions acted at the left and right edges. These tensions correspond to the contractile force per unit cross-sectional area in the unloaded configuration:

$${T}_{{\rm{SR}},i}={\gamma }_{L}{\dot{\lambda }}_{i}+{T}_{\psi L}({\lambda }_{i},{\mu }_{i})+{T}_{{\rm{act}},i}+{T}_{L{\rm{\max }}}({\lambda }_{i}),\,i=1,\cdots ,ns.$$

The first term represents the frictional tension, while *γ*_*L*_ is the friction coefficient. *T*_*ψL*_(*λ*_*i*_, *μ*_*i*_) is the passive contractile tension given in equation (4), *T*_act,*i*_ is the active contractile tension, and *T*_*L*max_ is the passive tension generated by titin (Fig. 1) to prevent extraordinary stretching of the half-sarcomere.

$${T}_{Lmax}(\lambda )=\{\begin{array}{cc}0, & \lambda \le {\lambda }_{Lmax}\\ {c}_{Lmax}{(\lambda -{\lambda }_{Lmax})}^{3}, & \lambda  > {\lambda }_{Lmax}\,\end{array}.$$

The values of *c*_*L*max_ = 100 MPa and *λ*_*L*max_ = 1.25 were used in the present study.

From the longitudinal mechanical equilibria at the interfaces of the half-sarcomeres, the following equations, with a fixed left end, must be fulfilled:

$$\{\begin{array}{c}\begin{array}{c}{T}_{{\rm{S}}{\rm{R}},i}-{T}_{{\rm{S}}{\rm{R}},i+1}=0,\,i=1,\cdots ,ns-1\\ {\gamma }_{LE}{\dot{L}}_{ns}+{k}_{LE}({L}_{ns}-\bar{L})-{T}_{{\rm{S}}{\rm{R}},ns}=0\end{array}.\end{array}$$

Here, *k*_*LE*_ is the spring constant per unit area of the spring connected to the right edge of the myofibril, *L*_*ns*_ is the total length of the myofibril, and $\bar{L}$ is the length of the myofibril at which the spring force is zero. In the present study, $\bar{L}$ was always set to a length 20% longer than its natural length in the unloaded condition. *γ*_*LE*_ is the friction coefficient suppressing edge movement, which was set to a nonzero value only for the IFM model. *k*_*LE*_ was set to *k*_*LE*_ = 20 [KPa/μm] for the standard and isotonic SPOC (*ns* = 40), or to *k*_*LE*_ = 0.08 [KPa/μm] for the IFM (*ns* = 5000).

The transversal mechanical equilibrium in each half-sarcomere was represented by:

$$\{\begin{array}{ccc}{\gamma }_{T}{\dot{\mu }}_{1}+{T}_{\psi T}({\lambda }_{1},{\mu }_{1})+{T}_{\phi ,1,2}({\mu }_{1},{\mu }_{2}) & = & 0\\ {\gamma }_{T}{\dot{\mu }}_{i}+{T}_{\psi T}({\lambda }_{i},{\mu }_{i})+{T}_{\phi ,i,i-1}({\mu }_{i},{\mu }_{i-1})+{T}_{\phi ,i.i+1}({\mu }_{i},{\mu }_{i+1}) & = & 0,\,i=2,\cdots ,ns-1\\ {\gamma }_{T}{\dot{\mu }}_{ns}+{T}_{\psi T}({\lambda }_{ns},{\mu }_{ns})+{T}_{\phi ,ns,ns-1}({\mu }_{ns},{\mu }_{ns-1}) & = & 0\end{array}.$$

Here, the first term is the transverse frictional tension with a frictional coefficient *γ*_*T*_. We assumed that the tension from the lattice space alignment *T*_*ϕ*_ comes only from one side at the two ends of the myofibril.

Our study used a parallel MPI-OpenMP hybrid computer system, and the computational time step size Δ*T* of the myofibril dynamics was given by an integer multiple of the time step size Δ*t *of the actomyosin dynamics (${\rm{\Delta }}T=nt\cdot {\rm{\Delta }}t$) simulated by a Monte Carlo (MC) approach to reduce the synchronization and communication overhead^19^. The time step sizes Δ*t* and Δ*T* were set to 2.5 μs and 62.5 μs (*nt* = 25), respectively, for the standard SPOC simulation, and to 0.5 μs and 5 μs (*nt* = 10), respectively, for the IFM simulation.

Below, we describe the algorithm used to couple the two scales. We previously reported the detailed derivation of this algorithm^4,19^. The variables of the system containing the myofibril were assigned to an unknown vector and its time derivative:

$${\boldsymbol{U}}=(\begin{array}{c}{\boldsymbol{L}}\\ {\boldsymbol{\mu }}\end{array}),\,\dot{{\boldsymbol{U}}}=(\begin{array}{c}\dot{{\boldsymbol{L}}}\\ \dot{{\boldsymbol{\mu }}}\end{array}).$$

Here, ***L*** consists of the positions of the half-sarcomere boundaries from which the longitudinal stretching parameter is given by:

$${\lambda }_{i}=2\frac{{L}_{i}-{L}_{i-1}}{S{L}_{0}}.$$

We assumed that the longitudinal and transversal equilibrium equations is represented by:

$${\boldsymbol{G}}({\boldsymbol{U}},\dot{{\boldsymbol{U}}})=(\begin{array}{c}{{\boldsymbol{G}}}_{L}({\boldsymbol{U}},\dot{{\boldsymbol{U}}})\\ {{\boldsymbol{G}}}_{t}({\boldsymbol{U}},\dot{{\boldsymbol{U}}})\end{array})=0.$$

In the time integration scheme, the variables ***U*** and $\dot{{\boldsymbol{U}}}$ were updated from those at time *T* to the time $T+{\rm{\Delta }}T$, so that the interpolation relationships and the equilibrium condition at time $T+{\rm{\Delta }}T$ were fulfilled:

$$\{\begin{array}{ccc}{}^{T+{\rm{\Delta }}T}{\boldsymbol{U}} & = & {}^{T}{\boldsymbol{U}}+{\rm{\Delta }}T({\omega }^{T+{\rm{\Delta }}T}\dot{{\boldsymbol{U}}}+{(1-\omega )}^{T}\dot{{\boldsymbol{U}}})\\ {\boldsymbol{G}}{(}^{T+{\rm{\Delta }}T}{\boldsymbol{U}}{,}^{T+{\rm{\Delta }}T}\dot{{\boldsymbol{U}}}) & = & 0\end{array}.$$

Here, ω is the interpolation weight that determined the numerical accuracy and stability of this scheme. In our case, we used *ω* = 0.6. The above equation was solved using Newton’s iteration with the derivatives ∂***G***/∂***U*** and $\partial {\boldsymbol{G}}/\partial \dot{{\boldsymbol{U}}}$. To compute the longitudinal term ${{\boldsymbol{G}}}_{L}{(}^{T+{\rm{\Delta }}T}{\boldsymbol{U}},{}^{T+{\rm{\Delta }}T}\dot{{\boldsymbol{U}}})$, we need to determine the active tension *T*_act_ at time $T+{\rm{\Delta }}T$ by performing MC simulations for individual actomyosin systems over the time interval [*T*, *T* + Δ*T*]. The MC simulations were performed by applying the longitudinal stretch rate at time *T* for temporarily computing the rod strain. In the Newton’s iteration at time *T* + Δ*T*, the active tension was given by:

$${}^{T+{\rm{\Delta }}T}{T}_{{\rm{a}}{\rm{c}}{\rm{t}}}=\frac{2}{S{A}_{0}\cdot nt\cdot {n}_{F}}\sum _{k=1}^{nt}\sum _{\beta =1}^{{n}_{F}}\sum _{\alpha =1}^{{n}_{M}}{}^{T+k{\rm{\Delta }}t}{\delta }_{A,\alpha ,\beta }\frac{d{W}_{rod}}{dx}({}^{T+k{\rm{\Delta }}t}{x}_{\alpha ,\beta }).$$

The rod strain was given by the summation of the initial strain at attachment *x*_*A*_, the total power-stroke distance *s*, and the increment caused by the half-sarcomere shortening distance Δ*z* from the most recent attachment, as follows.

$${}^{T+k{\rm{\Delta }}t}x={x}_{A}+{}^{T+k{\rm{\Delta }}t}s-{}^{T+k{\rm{\Delta }}t}{\rm{\Delta }}z.$$

To implicitly account for the feedback between the rod strain and the SL changes over the time interval [*T*, *T* + Δ*T*], the increment caused by the SL change was determined from the sarcomere stretch rate at time *T* + Δ*T*, as follows:

$${}^{T+k{\rm{\Delta }}t}{\rm{\Delta }}z=\{\begin{array}{cc}{}^{T}{\rm{\Delta }}z-k{\rm{\Delta }}t{\frac{S{L}_{0}}{2}}^{T+{\rm{\Delta }}T}\dot{\lambda }, & \,{t}_{A} < T\\ -(k-{k}_{A}){\rm{\Delta }}t{\frac{S{L}_{0}}{2}}^{T+{\rm{\Delta }}T}\dot{\lambda }, & {t}_{A}\ge T\,\end{array}.$$

Here, *t*_*A*_ was the most recent time at which the myosin molecule was bound.

## Supplementary information

Supplementary figures

Dataset 1

Computer code

## Data Availability

The software and experimental data are available in Supplementary Information. The software is released under the MIT License.
