# Supplementary material for: Effect of myofibril passive elastic properties on the mechanical communication between motor proteins on adjacent sarcomeres
Source: Sci Rep. 2019 Jun 27;9:9355. doi: 10.1038/s41598-019-45772-1 (PMC6597731; doi:10.1038/s41598-019-45772-1)
Supplement: Supplementary file 1 — Supplementary figures [file 41598_2019_45772_MOESM1_ESM.pdf]

Supplemental Figures

Effect of myofibril passive elastic properties on the mechanical communication between motor proteins on adjacent sarcomeres

Takumi Washio*[1], Seine A. Shintani[2], Hideo Higuchi[2], Seiryo Sugiura[1] & Toshiaki Hisada[1]

[1]UT-Heart Inc. Future Center Initiative, University of Tokyo, 178-4-4 Wakashiba, Kashiwa 277-0871, Japan

[2]Department of Biomedical Sciences, College of Life and Health Sciences, Chubu University, 1200 Matsumoto-cho Kasugai, Aichi 487-8501, Japan

[3]Graduate School of Science, University of Tokyo, 7-3-1 Hongo Bunkyo-ku, Tokyo 113-0033, Japan

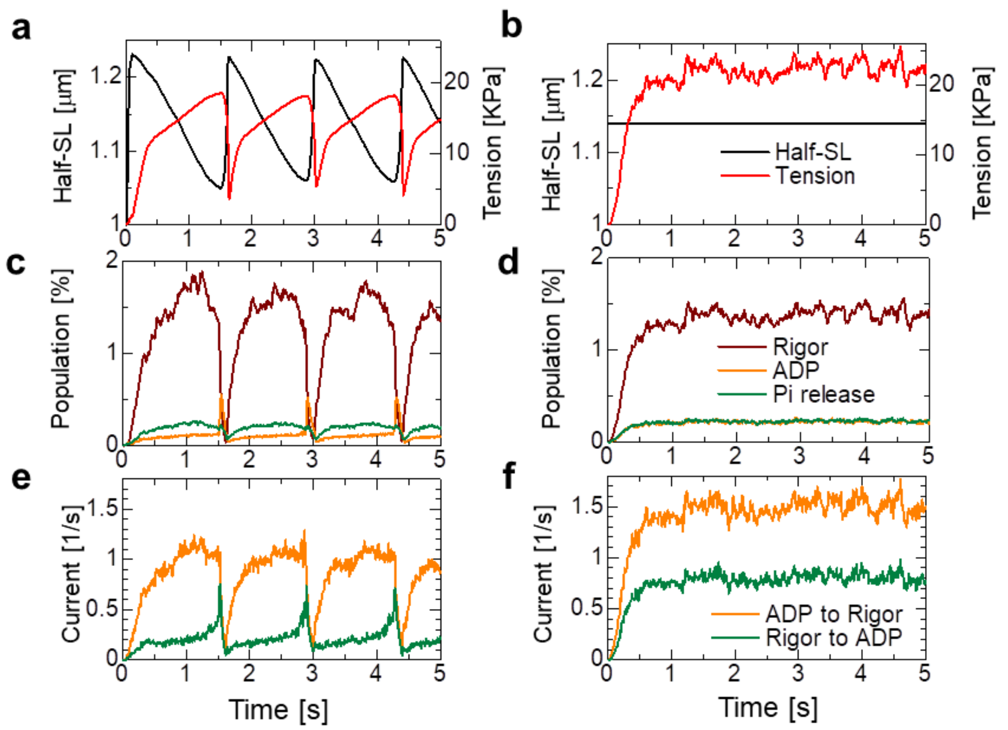

Figure S1. The numerical results from the single half-sarcomere model. In (**a, c, e**), the half-sarcomere is connected with the external spring. The parameters for the passive properties were $k_{MM} = 1$ MPa, $k_{MZ} = 0.5$ MPa, $k_{LT} = 0.1$ MPa, $k_{LL} = 0.01$ MPa, and $k_{TT} = 0.1$ MPa. The spring constant of the external spring was $k_L = 20$ [KPa/ μm]. In (**b, d, f**), the half-SL was fixed, and thus the active tension is solely determined from the crossbridge dynamics. (**a, b**) Time transients of the half-SL (black) and the active tension (red). (**c, d**) The time transients of populations of the bound states (Pi release, green; ADP, orange; Rigor, brown). Because the applied Ca^{2+} concentration ([Ca] = 0.2 μM) is an intermediate concentration value for activation, the populations of bound states are relatively small. (**e, f**) The time transients of the state transition currents between the ADP state and the Rigor state. The power-stroke currents are coloured orange, while the reversal power-stroke currents are coloured green. In cases with an

external spring, a rapid increase in ADP state (**c**) is observed, along with an increase in reversal power-stroke current (**e**) and the avalanche of reversal power-strokes.

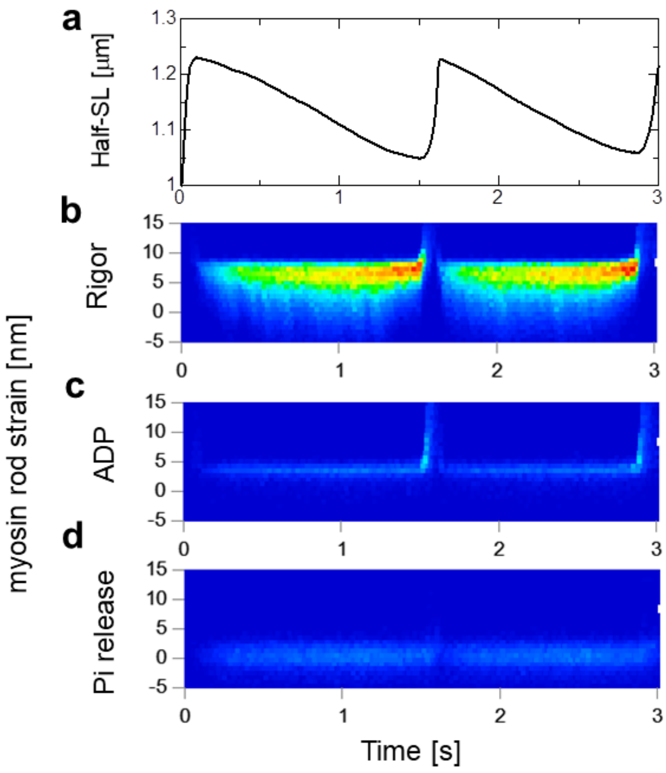

Figure S2. Numerical results from the single half-sarcomere model connected with the external spring. The parameters for the passive properties were $k_{MM} = 1$ MPa, $k_{MZ} = 0.5$ MPa, $k_{LT} = 0.1$ MPa, $k_{LL} = 0.01$ MPa, and $k_{TT} = 0.1$ MPa. The spring constant of the external spring was $k_{LE} = 20$ [KPa/μm]. The contours of the time course of the densities with respect to the myosin rod strain for the Rigor state (**b**), the ADP state (**c**), and Pi release state (**d**) are depicted along with the time transients of the half-SL (**a**). In the Rigor state (**b**), the centre of the distribution shifted to the larger rod strains towards when sarcomere shortening slows down. Such a distribution shift has a major impact on balancing the frequency of the power-strokes and the reversal power-strokes determined by equation (9). See also Supplementary Fig. S1e.

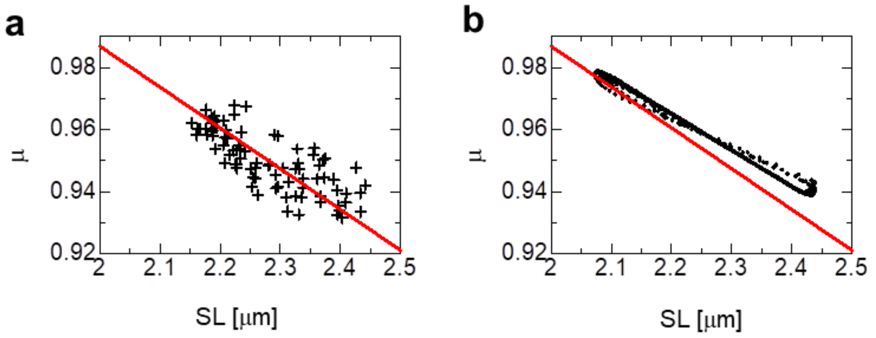

Figure S3. Plots of the SL-LS relationship for SPOC using the experimental results from the rabbit iliopsoas muscle (**a**) and the numerical results from the standard SPOC model (**b**). The red lines represent the SL-LS relationship given by $R(\lambda, \mu) = \lambda - 1 + 2\beta_R(\mu - 1) = 0$, with $\beta_R = 2$. For the experimental results (**a**), the stretching parameter μ in the transverse direction was found by assuming that the unloaded A-band width was equal to 1.26 μm.

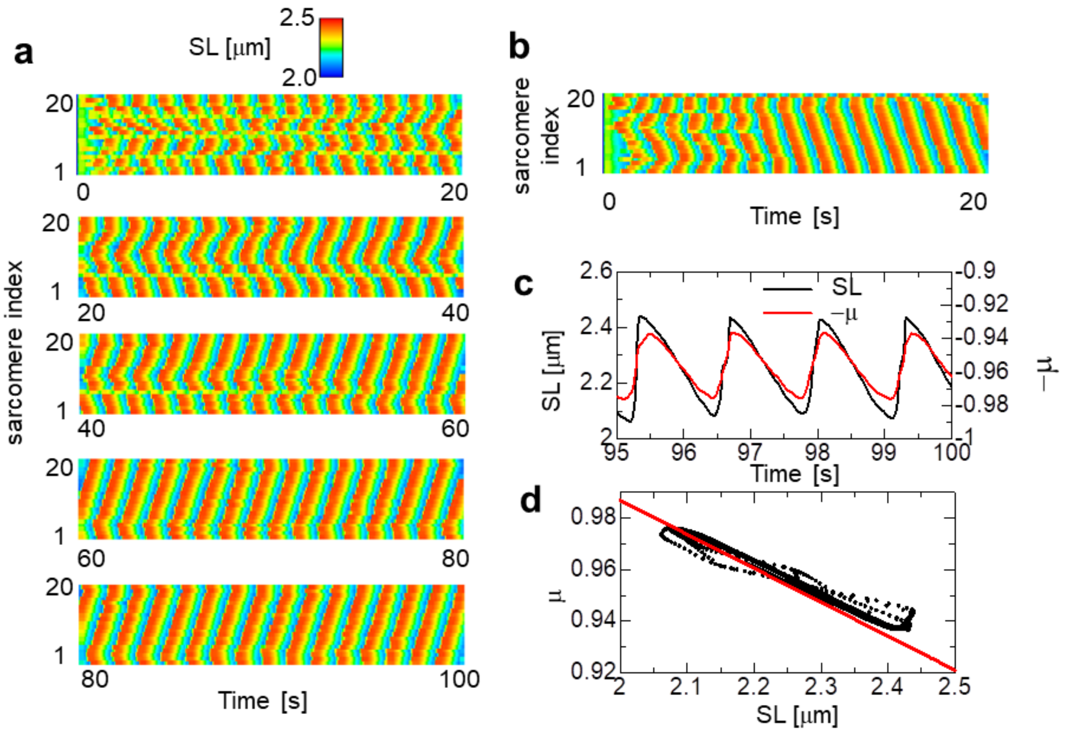

Figure S4. Numerical results of the standard SPOC model using a weaker stiffness for the inverse SL-LS relationship (k_{LT}) and the transverse stretching (k_{TT}). The parameters for the passive properties were $k_{MM} = 1$ MPa, $k_{MZ} = 0.5$ MPa, $k_{LT} = 0.025$ MPa, $k_{LL} = 0.01$ MPa, and $k_{TT} = 0.01$ MPa. (**a**) The spatiotemporal pattern of the SL from 0 s to 100 s. The pattern starts with out-of-phase synchronization (0 s–20 s), which then became a disrupted traveling wave (20 s–60 s), and finally settled into a traveling wave (60 s–100 s). (**b**) The spatiotemporal pattern of the SL from 0 s to 20 s for the standard model, in which $k_{LT} = 0.1$ MPa and $k_{TT} = 0.1$ MPa. (**c**) The time transients of the SL (black) and the transverse stretching parameter μ of the sarcomere consisting of #19 and #20 half-sarcomeres. The negative of μ is plotted to show the relationship with the SL. (**d**) Plots of the SL-LS relationship in (**b**). The red lines indicate the SL-LS relationship represented by $R(\lambda, \mu) = \lambda - 1 + 2\beta_R(\mu - 1) = 0$ with $\beta_R = 2$.

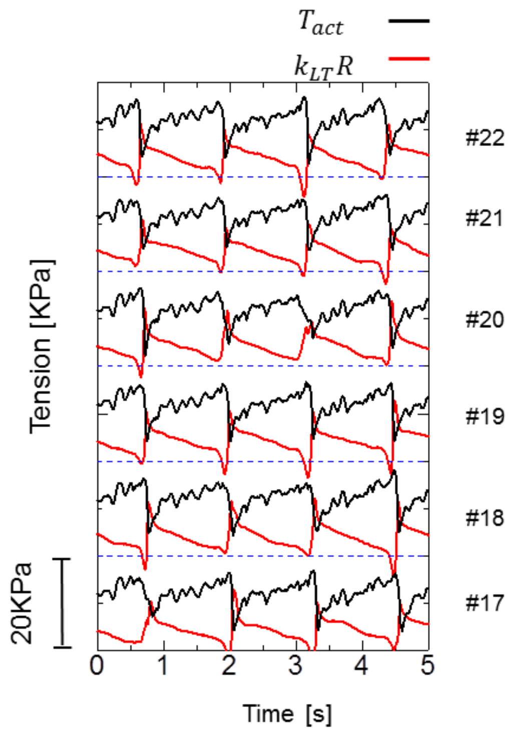

Figure S5. Numerical results of the SPOC model using auxotonic boundary conditions. The time transients of the active tension (T_{act}: black) and the longitudinal passive force ($k_{LT}R$: red) generated by the inverse SL-LS relationship are superposed for the half sarcomeres from #17 to #22. The fluctuations of active tensions were caused by changes in the tension at the myofibril boundary. Although the profiles of the active tensions were irregular, all of them were similar.

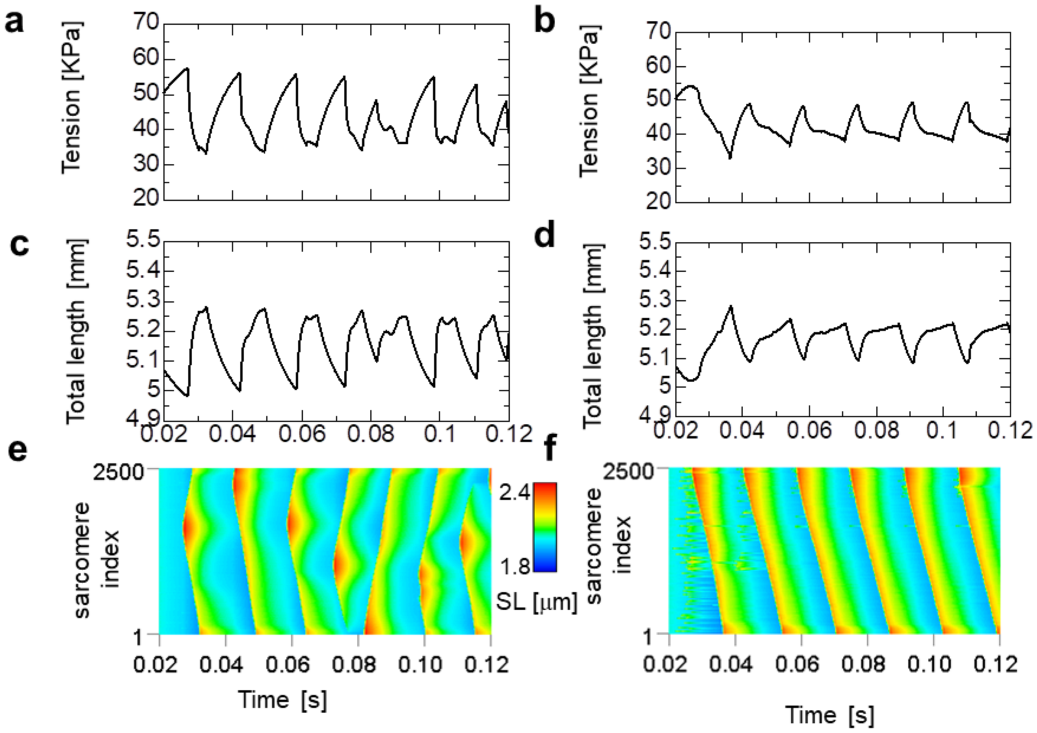

Figure S6. Numerical results of SPOC in the insect flight myofibril model consisting of 5,000 half-sarcomeres using the weaker stiffness for the lattice alignment (left: $k_{MM} = 25$ GPa, $k_{MZ} = 12.5$ GPa), and with weaker stiffness for the inverse SL-LS relationship (right: $k_{LT} = 0.02$ MPa). (**a, b**) The time transient of the tension generated at the free end of the myofibril, in which resistance with a friction coefficient $\gamma_{LE} = 20\mathrm{Pa \cdot s/mm}$ was imposed. (**c, d**) The time transient of the total myofibril length. (**e, f**) The spatiotemporal pattern of SL for all of the sarcomeres.

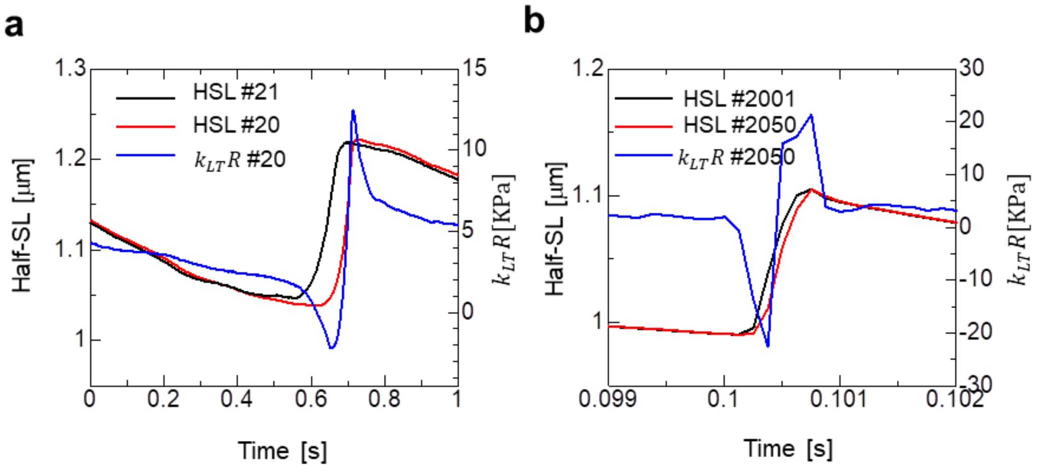

Figure S7. Time transients of the half-LSs and the passive tensions from the inverse SL-LS relationship ($k_{LT}R(\lambda, \mu)$) during half-sarcomere lengthening. (**a**) The time transients of the half-SLs of #20 and #21 in the standard SPOC model, in which the lengthening wave propagated from #40 to #1 (Fig. 4). The blue line is the passive tension from the inverse SL-LS relationship at the half-sarcomere #20. The drop in this passive tension starts at the same time as the rise in the half-SL of the adjacent upstream half-sarcomere #21 (black line). The rise in the half-SL of #20 (red line) started after the half-SL of #20 reached its maximal value. (**b**) The time transients of the half-SLs of #2001 and #2050 in the IFM model, for which the lengthening wave propagated from #1 to #5000 (Fig. 9). The blue line is the passive tension from the inverse SL-LS relationship for the half-sarcomere #2500. The drop of this passive tension started earlier than the rise in the half-SL of #2001 (black line), which was located approximately 50 half-sarcomeres away in the upstream direction from #2050. Further, the increase in the half-SL of #2050 (red line) started much earlier than the completion

of lengthening of #2001 (black line). These features of wave propagation differed markedly from those observed in the standard SPOC model.

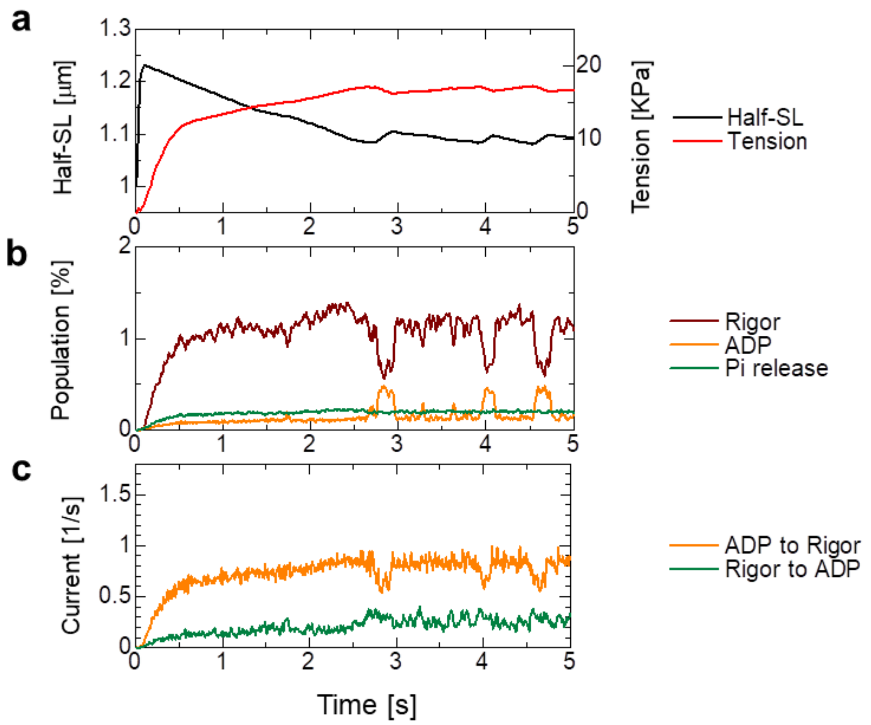

Figure S8. Numerical results from the single half-sarcomere model. Except for the upper limits of the reversal power-power stroke transition rate constants ($\bar{b}_1$, $\bar{b}_2$), all the parameters were set to the same values as those in Supplemental Fig. S1. The upper limits were set as $\bar{b}_1 = \bar{b}_2 = 62.5$ [1/s], which are half of the values of the standard SPOC model. (**a**) Time transients of the half-SL (black) and the active tension (red). (**b**) The time transients of populations of the bound states (Pi release, green; ADP, orange; Rigor, brown). Because the applied Ca^{2+} concentration ([Ca] = 0.2 μM) is an intermediate concentration value for activation, the populations of the bound states are relatively small. (**c**) The time transients of the state transition currents between the ADP state and the Rigor state. The power-stroke currents are coloured orange, while the reversal power-stroke currents are coloured green. Compared with the numerical results

in Supplemental Fig. S1, the oscillatory behaviour almost disappeared. In particular, the increase of the transition current from the Rigor state to the ADP state was lost (**c**).
